# Ubiquitin–proteasome system in the different stages of dominantly inherited Alzheimer's disease

**DOI:** 10.1002/alz.70243

**Published:** 2025-05-24

**Authors:** Haiyan Liu, Quoc Bui, Jason Hassenstab, Brian A. Gordon, Tammie L. S. Benzinger, Jigyasha Timsina, Yun Ju Sung, Celeste Karch, Alan E. Renton, Alisha Daniels, John C. Morris, Chengjie Xiong, Laura Ibanez, Richard J. Perrin, Jorge J. Llibre‐Guerra, Gregory S. Day, Charlene Supnet‐Bell, Xiong Xu, Sarah B. Berman, Jasmeer P. Chhatwal, Takeshi Ikeuchi, Kensaku Kasuga, Yoshiki Niimi, Edward D. Huey, Peter R. Schofield, William S. Brooks, Natalie S. Ryan, Mathias Jucker, Christoph Laske, Johannes Levin, Jonathan Vöglein, Jee Hoon Roh, Francisco Lopera, Randall J. Bateman, Carlos Cruchaga, Eric M. McDade, For DIAN study team

**Affiliations:** ^1^ Department of Neurology Washington University in St. Louis St. Louis Missouri USA; ^2^ Department of Biostatistics Washington University in St. Louis St. Louis Missouri USA; ^3^ Department of Radiology Washington University in St. Louis St. Louis Missouri USA; ^4^ Department of Psychiatry Washington University in St. Louis St. Louis Missouri USA; ^5^ Department of Genetics and Genomic Sciences Icahn School of Medicine at Mount Sinai New York New York USA; ^6^ Department of Pathology and Immunology Washington University in St. Louis St. Louis Missouri USA; ^7^ Department of Neurology Mayo Clinic in Florida Jacksonville Florida USA; ^8^ Departments of Neurology and Clinical & Translational Science University of Pittsburgh Pittsburgh Pennsylvania USA; ^9^ Brigham and Women's Hospital, Massachusetts General Hospital Harvard Medical School Cambridge Massachusetts USA; ^10^ Brain Research Institute Niigata University Niigata Japan; ^11^ Specially appointed lecturer, Unit for Early and Exploratory Clinical Development The University of Tokyo Tokyo Japan; ^12^ Memory and Aging Program, Butler Hospital, Department of Psychiatry and Human Behavior, Alpert Medical School Brown University Providence Rhode Island USA; ^13^ Neuroscience Research Australia Sydney New South Wales Australia; ^14^ Faculty of Medicine and Health University of New South Wales Sydney New South Wales Australia; ^15^ Dementia Research Centre UCL Queen Square Institute of Neurology London UK; ^16^ UK Dementia Research Institute at UCL London UK; ^17^ German Center for Neurodegenerative Diseases (DZNE) Tübingen Tübingen Germany; ^18^ Section for Dementia Research, Hertie Institute for Clinical Brain Research, Department of Psychiatry and Psychotherapy University of Tübingen Tübingen Germany; ^19^ German Center for Neurodegenerative Diseases Site Munich Munich Germany; ^20^ Department of Neurology Ludwig‐Maximilians‐Universität München Munich Germany; ^21^ Munich Cluster for Systems Neurology (SyNergy) Munich Germany; ^22^ Department of Neurology LMU University Hospital LMU Munich Munich Germany; ^23^ German Center for Neurodegenerative Diseases (DZNE) Munich Germany; ^24^ Departments of Neurology and Physiology Korea University Anam Hospital Korea University College of Medicine Seoul South Korea; ^25^ Grupo de Neurociencias de Antioquia (GNA), Facultad de Medicina Universidad de Antioquia Medellín Colombia

**Keywords:** amyloid beta, amyloid precursor protein, autophagy–lysosome pathway, biomarker discovery, dominantly inherited Alzheimer's disease, genetic mutations, neurodegeneration, presenilin 1, presenilin 2, protein aggregation, protein degradation, proteomic analysis, proteostasis, tau pathology, ubiquitin–proteasome system

## Abstract

**INTRODUCTION:**

This study investigated the role of the ubiquitin–proteasome system (UPS) in dominantly inherited Alzheimer's disease (DIAD) by examining cerebrospinal fluid (CSF) levels of UPS proteins.

**METHOD:**

The SOMAscan assay was used to detect changes in UPS proteins in mutation carriers (MCs) relative to disease progression; imaging and CSF biomarkers of amyloid, tau, and neurodegeneration measures; and Clinical Dementia Rating scale.

**RESULTS:**

Subtle increases in specific ubiquitin enzymes were detected in MCs up to two decades before symptom onset, with more pronounced elevations in UPS‐activating enzymes near symptom onset. Significant correlations were found between UPS proteins and Alzheimer's disease (AD) biomarkers, especially between autophagy markers and late‐stage tau biomarkers, microglia, and axonal degeneration.

**DISCUSSION:**

The rise in UPS proteins alongside tau‐related markers suggests UPS involvement in tau neurofibrillary tangles. Elevated CSF UPS proteins in DIAD MCs may serve as indicators of disease progression, and may support the UPS as a therapeutic target in AD.

**Highlights:**

- This study investigates the ubiquitin‐proteasome system (UPS) in Dominantly Inherited Alzheimer's Disease (DIAD), highlighting early molecular changes linked to disease progression.
- Using SOMAscan proteomics, we identified significant UPS protein alterations in cerebrospinal fluid of mutation carriers, notably up to 20 years before clinical symptom onset.
- Correlations between UPS protein levels and Alzheimer's biomarkers, particularly tau and neurodegeneration markers, suggest a strong association between UPS dysregulation and tau pathology in DIAD.
- Dynamic UPS changes align with A/T biological staging: UPS proteins were shown to increase across Aβ/tau (A/T) groups, with largest increases in the A+/T+ group, reinforcing their role in late‐stage tau pathology and disease progression.
- These findings underscore the potential of UPS proteins as early biomarkers for Alzheimer's disease progression and as novel therapeutic targets, especially in tau‐pathology‐driven neurodegeneration.
- This work contributes to understanding AD pathogenesis, by emphasizing the importance of protein quality control systems and by offering avenues for future biomarker discovery and therapeutic development in Alzheimer's disease.

## 1 BACKGROUND

Alzheimer's disease (AD) is a multifactorial disorder influenced by a variety of genetic and environmental factors.^1^ The disease is characterized by the accumulation of misfolded, insoluble protein aggregates, composed primarily of amyloid beta (Aβ) peptide (plaques), and phosphorylated tau protein (forming neurofibrillary tangles [NFTs]) in the brain,^2^ which leads to the insidious onset and gradual disruption of cognitive and behavioral functions.^2^, ^3^, ^4^

Recent studies highlight the role of faulty proteostasis in the progression of neurodegenerative diseases.^5^ Proteostasis encompasses cellular mechanisms that regulate protein synthesis, folding, post‐translational modification, and degradation, mechanisms that are disrupted in conditions like AD.^6^ The ubiquitin–proteasome system (UPS) and the autophagy–lysosomal pathway work in tandem to preserve proteostasis in cells by preventing the accumulation of non‐functional and misfolded proteins.^7^, ^8^ UPS degrades substrates that are potentially toxic by breaking them down into small peptides to replenish intracellular amino acid pools.^9^ In humans, the UPS consists of two activating enzymes (E1s), ≈ 40 conjugating enzymes (E2s), > 600 ligase enzymes (E3s), and ≈ 100 deubiquitinases (DUBs).^10^, ^11^ Proteostasis defects can lead to neuronal stress, synapse loss, and memory deficits, such that impaired proteostasis is considered a main contributor to AD pathogenesis.^5^

The association between proteasomal dysfunction and AD was first established through histopathological examinations, which highlighted the accumulation of ubiquitin in AD‐associated plaques and tangles.^12^, ^13^ Subsequent genome‐wide association studies (GWAS) and proteomic studies have corroborated this link by identifying key roles for the proteasomal pathway in patients with symptomatic AD and transgenic AD models.^12^, ^14^, ^15^, ^16^ These advanced methodologies uncovered significant changes at the proteome level during AD progression, particularly highlighting the dysregulation of the UPS.^16^ This dysregulation is characterized by changes in the levels of certain ubiquitin‐activating and ubiquitin‐conjugating enzymes that can lead to the inhibition of proteasome activity.^17^, ^18^ Additionally, the dysregulation of ubiquitin‐mediated pathways is associated with alterations in learning and memory ability, Aβ plaque formation, hyperphosphorylation of tau protein, as well as synaptic plasticity and immune function changes in AD mouse models.^12^, ^13^ The potential therapeutic implications of these findings are underscored by the promising effects of small molecules targeting the proteasomal pathway in animal and cellular models of AD.^19^ To date, most studies of the UPS have been undertaken using animal or cellular models of AD or in brain tissue of symptomatic AD cases. Given the recent evolution of methods for studying AD pathology biomarkers in humans, there is now the opportunity to evaluate the role of the UPS system in the presymptomatic and symptomatic stages of AD.

Studies in dominantly inherited Alzheimer's disease (DIAD) allow the examination of disease‐related proteins from the presymptomatic stage to moderately symptomatic stages of AD over three decades of disease progression. Here we analyzed cerebrospinal fluid (CSF) proteomic data from DIAD individuals. Leveraging the high‐throughput capabilities of the SOMAscan proteomics platform and data from the Dominantly Inherited Alzheimer Network (DIAN), we explored the changes in expression, stability, and modifications of UPS proteins throughout the disease course. Considering existing evidence that abnormal accumulation of Aβ and tau proteins in the brain in AD begins well before the onset of neurological symptoms, up to 20 years prior, we investigated the early accumulation of both Aβ and tau aggregated protein species in relation to UPS dysregulation in DIAD.^18^, ^20^, ^21^ We aimed to explore if dysregulation of UPS proteins impacts the progression of DIAD by assessing the associations with Aβ and tau pathologies, neuronal loss, and neuroinflammation (all measured using existing established CSF and neuroimaging biomarkers) and clinical symptoms. Our findings could provide important insights into AD initiation and progression and potentially reveal novel biomarkers of disease progression and new therapeutic targets.

## 2 METHODS

### 2.1 Participants

The DIAN observational study (DIAN Obs) recruited participants from families that carry an autosomal‐dominant AD mutation in one of three genes—*APP, PSEN1*, or *PSEN2*. DIAN Obs is a longitudinal, observational study in which participants undergo comprehensive assessments including clinical and neuropsychological testing, brain imaging, and collection of biofluids such as CSF and blood.^22^, ^23^ This analysis incorporated cross‐sectional clinical data and CSF measures in 289 mutation carriers (MCs) and 172 mutation non‐carrier participant controls (NCs) from data freeze 15, each with at least one CSF measure.^24^ Mutation status was determined using polymerase chain reaction–based amplification of the relevant exon(s) followed by Sanger sequencing.^20^

To ensure participant confidentiality and due to the limited number of individuals at the extreme ends of the timeline, we have not displayed individual participant data for the period before –30 years and after 10 years of estimated symptom onset.

**RESEARCH IN CONTEXT**

1. **Systematic review**: This study investigates the ubiquitin‐proteasome system (UPS) in Dominantly Inherited Alzheimer's Disease (DIAD). The UPS is crucial for protein degradation and proteostasis, and its dysfunction is implicated in neurodegenerative diseases like Alzheimer's. Using the SOMAscan proteomics platform, the study explores UPS protein levels in DIAD from presymptomatic to symptomatic stages, linking them to amyloid, tau, and neurodegeneration biomarkers.
2. **Interpretation**: The results show that UPS proteins increase up to 20 years before symptom onset, correlating with amyloid and tau markers. Specifically, UPS proteins like UBE2N and SMURF1 are linked to tau pathology, suggesting that UPS dysfunction plays a role in tau aggregation and neurodegeneration. These findings position UPS as an early marker of DIAD progression, with potential therapeutic implications.
3. **Future directions**: Future research should include longitudinal studies to clarify the timing of UPS dysregulation in relation to disease onset and tau pathology. Expanding UPS protein analysis, especially related to autophagy, and exploring clinical trials targeting UPS dysfunction could provide new biomarkers and therapeutic strategies for AD.

### 2.2 CSF sample collection and protein measurements by SOMAscan

CSF samples were collected after an overnight fast and preserved at –80°C for subsequent protein level measurements using the Slow Off‐rate Aptamer (SOMAmer)‐based capture array, SOMAscan. Protein measurements reported in relative fluorescence units (RFUs) underwent hybridization, median, and iterative adaptive normalization by maximum likelihood (ANML) procedures until convergence. Ensuring data integrity, we performed an in‐house quality control, excluding aptamers shared by ≈ 70% of participant sample outliers.^25^

All proteins of interest were analyzed using the SOMAscan assay (v4.1) from SomaLogic, which profiles ≈ 7000 proteins.^25^ To identify UPS proteins within our SOMAscan dataset, the UniProt representational state transfer (REST) application programming interface (API) was used to cross‐reference our dataset with the UPS category in UniProt's controlled vocabulary. Further refinement was achieved using the fetching annotations from UniProt and Reactome databases. Discrepancies were manually verified for accuracy. We identified 174 UPS proteins from a SOMAscan pool of ≈ 6600 proteins for further analysis.

### 2.3 Immunoassay

The levels of Aβ42, Aβ40, and total tau (t‐tau) in CSF were measured using the Lumipulse platform (Fujirebio) through immunoassay techniques.^26^ Phosphorylated to unphosphorylated ratio of tau at threonine 181, 205, and 217, serine 202 (pT181/T181, pT205/T205, pT217/T217, and pS202/S202) in CSF were measured by mass spectrometry.^27^, ^28^ CSF soluble triggering receptor expressed on myeloid cells 2 (sTREM2) immunoassay was performed as described previously.^26^ Neurofilament light chain (NfL) levels were measured in CSF and serum using enzyme‐linked immunosorbent assay (ELISA).^29^, ^30^

### 2.4 Clinical assessment and DIAN estimated year from symptom onset

The Clinical Dementia Rating–Sum of Boxes (CDR‐SB) assessment scale was used to assess the stage of dementia in a blinded manner by clinical evaluators (the scale ranges from 0 to 18, with higher scores denoting more significant impairment).^31^ The participant's estimated years from symptom onset (EYO) was calculated at each visit based on their age and expected age of symptom onset specific to their mutation. If this information was unavailable, the EYO was calculated at the age at which parental cognitive decline began, as determined through a semi‐structured interview and historical data.^32^

### 2.5 Imaging

Imaging included magnetic resonance imaging (MRI) and positron emission tomography (PET) imaging for volumetric analyses as well as evaluations of Aβ (using ^11^C‐Pittsburgh Compound B [PiB] PET) and glucose metabolism (using 18F‐fluorodeoxyglucose [FDG] PET) as detailed previously.^23^ Using FreeSurfer 5.3, we defined cortical and subcortical regions of interest (ROIs). Both PET modalities were partially volume corrected via a regional spread function technique.^29^, ^33^ Our study concentrated on the precuneus region for its early and consistent involvement by AD pathology in DIAD.^20^, ^33^, ^34^ Tau PET imaging used 18F‐AV‐1451 (flortaucipir), with data from the 80 to 100 minute window converted to standardized uptake value ratios (SUVRs). To address differences in scanner spatial resolutions, scanner‐specific spatial filters were applied, standardizing to a common resolution of 8 mm ROI. PET data were also converted to SUVRs using the cerebellar gray matter as a reference. Partial volume correction was implemented using a regional spread function for each region, forming a geometric transfer matrix.^35^ The number of subjects contributing imaging data, including subsets for specific modalities, can be found in Table 1, clarifying the sample size across different imaging and CSF data points.

**TABLE 1:** Cohort demographics and fluid biomarkers.

	Group			
	NC (*n* = 172)	AsymMC (*n* = 179)	SymMC (*n* = 104)	*p* value
Female sex (*N* [%])	103 (59.9%)	96 (53.6%)	58 (55.8%)	0.79
*APOE* ε4 status positive (*N* [%])	57 (33.1%)	52 (29.1%)	29 (27.95)	0.96
Age (mean/SD/N)	39.22 (11.39)/172	35.57 (8.62)/179	47.39 (9.02)/104	<0·0001^*^; 0.002^†^
DIAN_EYO (mean/SD/N)	−9.04 (12.16)/172	−13.40 (8.58)/179	4.14 (2.89)/104	<0·0001^[Link]^, ^†^
Education (mean/SD/N)	15.01 (2.52)/126	15.00 (2.92)/130	13.25 (2.9)/84	<0.001^*^
CDR‐SB (mean/SD/N)	0.01 (0.05)/172	0.03 (0.12)/179	4.68 (4.22)/104	<0.001^*^
Cortical PiB PET SUVR (mean/SD/N)	1.07 (0.09)/136	1.30 (0.29)/137	1.75 (0.5)/72	<0·0001^[Link]^, ^†^
Precuneus FDG PET SUVR (mean/SD/N)	1.39 (0.1)/128	1.38 (0.11)/141	1.13 (0.21)/76	<0·0001^*^
Cortical tau PET SUVR (mean/SD/N)	1.21 (0.17)/11	1.39 (0.50)/12	5.78 (0.39)/3	<0·0001^*^
MRI precuneus thickness (mm3) left (mean/SD/N)	2.36 (0.14)/158	2.37 (0.15)/166	2.03 (0.27)/85	<0·0001^*^
MRI precuneus thickness (mm3) right (mean/SD/N)	2.39 (0.15)/158	2.38 (0.15)/166	2.08 (0.25)/85	<0·0001^*^
LUMIPULSE_CSF_Aβ42/40 ratio (mean/SD/N)	0.08 (0.03)/170	0.08 (0.03)/179	0.08 (0.03)/99	<0.001^*^
LUMIPULSE_CSF_Aβ40 (mean/SD/N)	9002.69 (2758.90)/169	8441.82 (3024.37)/177	7754.74 (2525.14)/102	<0.001^*^
LUMIPULSE_CSF_Aβ42 (mean/SD/N)	830.40 (286.30)/169	669.06 (357.66)/177	370.82 (180.75)/102	<0·0001^[Link]^, ^†^
LUMIPULSE_CSF_t‐au (mean/SD/N)	263.74 (108.52)/140	351.99 (220.42)/159	762.57 (382.94)/94	<0·0001^*^;0.005^†^
pT181/T181 (mean/SD/N)	21.38 (2.06)/99	26.13 (7.34)/100	33.95 (7.49)/68	<0·0001^[Link]^, ^†^
pT205/T205 (mean/SD/N)	0.34 (0.12)/99	0.43 (0.21)/100	0.96 (0.36)/68	<0·0001^*^;0.029^†^
pS202/S202 (mean/SD/N)	3.05 (0.73)/99	2.76 (0.88)/100	2.48 (0.64)/68	<0·0001^*^;0.022^†^
pT217/T217 (mean/SD/N)	1.23 (0.62)/99	3.33 (2.74)/100	8.76 (4.11)/68	<0·0001^[Link]^, ^†^
MTBR‐tau243 (mean/SD/N)	0.20 (0.08)/69	0.24 (0.11)/73	0.94 (0.69)/40	<0·0001^*^
log_CSF_NfL (mean/SD/N)	6.54 (0.44)/37	6.47 (0.42)/31	7.66 (0.80)/33	<0·0001^*^
Serum_NfL_log (mean/SD/N)	1.32 (0.22)/74	1.35 (0.19)/80	1.65 (0.26)/58	<0·0001^*^
sTREM2 (mean/SD/N)	0.49 (0.22)/77	0.44 (0.22)/57	0.72 (0.28)/63	<0·0001^*^

*N* = number.

*Notes*: Demographic and fluid biomarker data were presented as mean (SD) or *N* (%). For demographic analysis, *p* values were calculated using ANOVA based on raw data. Clinical Dementia Rating scores were adjusted for age and education and analyzed using ANCOVA based on raw data, with corresponding *p* values reported. Categorical variables such as sex and *APOE* status were analyzed using the χ2 test. Biochemical markers were analyzed using ANCOVA, considering log‐transformed variables and adjusting for age and sex. Hippocampal volume was corrected per participant by total brain volume. Certain *p* values were not specifically footnoted and were derived from ANOVA or ANCOVA comparisons. p‐Tau indicates phosphorylated tau on threonine 181, 205, 217, and serine 202. Notably, symptomatic carriers of pathogenic variants were compared to non‐carriers and asymptomatic carriers, with *p* < 0.0001 indicating statistical significance. Additional *p* values and comparisons were provided for various groups based on pathogenic variants, symptomatic carriers, non‐carriers, and asymptomatic carriers.

Abbreviations: Aβ, amyloid beta; ANCOVA, analysis of covariance; ANOVA, analysis of variance; *APOE*, apolipoprotein E; CSF, cerebrospinal fluid; DIAN, Dominantly Inherited Alzheimer's Network; EYO, estimated years to symptom onset; FDG, fluorodeoxyglucose; MC, mutation carrier; MRI, magnetic resonance imaging; NC, non‐mutation carrier; NfL, neurofilament light chain; PET, positron emission tomography; PiB, Pittsburgh compound B; SD, standard deviation; sTREM2, soluble triggering receptor expressed on myeloid cells 2; SUVR, standardized uptake value ratio; t‐tau, total tau.

^*^Symptomatic mutation carriers versus non‐ mutation carriers and asymptomatic mutation carriers, *p* < 0.0001.

^†^ Asymptomatic mutation carriers versus non‐mutation carriers, *p* < 0.0001.

### 2.6 Statistical analysis

In our study, cross‐sectional analyses were conducted to examine the descriptive characteristics and baseline biomarker values across distinct clinical groups. These analyses used chi‐squared (χ^2^) tests to assess differences in categorical variables and analysis of variance (ANOVA). This approach facilitated a detailed investigation of baseline biomarker discrepancies among the groups. Furthermore, we categorized mutation carrier participants into two distinct cohorts: asymptomatic carriers (those with a baseline CDR score of 0) and symptomatic carriers (those with a baseline CDR score > 0).

The cross‐sectional relationship of different levels of UPS protein between the two mutation groups along the DIAN EYO was evaluated using a linear mixed‐effects (LME) model. This model included fixed effects of the mutation group, EYO, and the interaction between mutation groups and EYO, along with random intercepts at the family level. Subsequently, a comparison of the estimated UPS levels between the two groups at each value of DIAN EYO, ranging from –30 to +10, was conducted. The EYO point at which the differences became statistically significant was determined by contrasting with specific EYO points. These estimators were then plotted against baseline EYO using local regression (LOESS).

Partial correlation analysis adjusted for age was conducted to assess the correlation between UPS proteins and each biomarker in each mutation group. Estimated correlation coefficients were compared using Fisher's *Z* transformation. Because of the large number of pairwise correlations to be compared, we controlled the false discovery rate (FDR) at the 5% level.^36^ Additionally, analysis of covariance (ANCOVA) for continuous variables was used to assess the differences between the NC and MC groups, taking age, sex, and apolipoprotein E (*APOE*) ε4 status into account as covariates, while also maintaining the FDR control at the 5% level.

Statistical analyses were performed using SAS version 9.4 (SAS Institute) and plots were created with RStudio (version 4.3.1). *P* values were obtained through two‐tailed tests, adopting a significance threshold of *p* < 0.05 to determine statistical significance.

## 3 RESULTS

### 3.1 Participant demographics

The cross‐sectional cohort study included 179 asymptomatic MCs with an average age of 35.6 years (standard deviation [SD] = 8.6) and an EYO of –13.4 years (SD = 8.6), 104 symptomatic MCs with an average age of 47.4 years (SD = 9.0) and an EYO of 4.1 years (SD = 2.9), and 172 asymptomatic mutation NCs with an average age of 39.2 years (SD = 11.4). The NCs were, on average, 9.0 years (SD = 12.2) younger relative to the EYO of their MC siblings. Comprehensive demographic details and baseline characteristics of the participants, as well as fluid and imaging biomarkers, are summarized in Table 1.

### 3.2 UPS protein changes in CSF

**FIGURE 1 alz70243-fig-0001:**
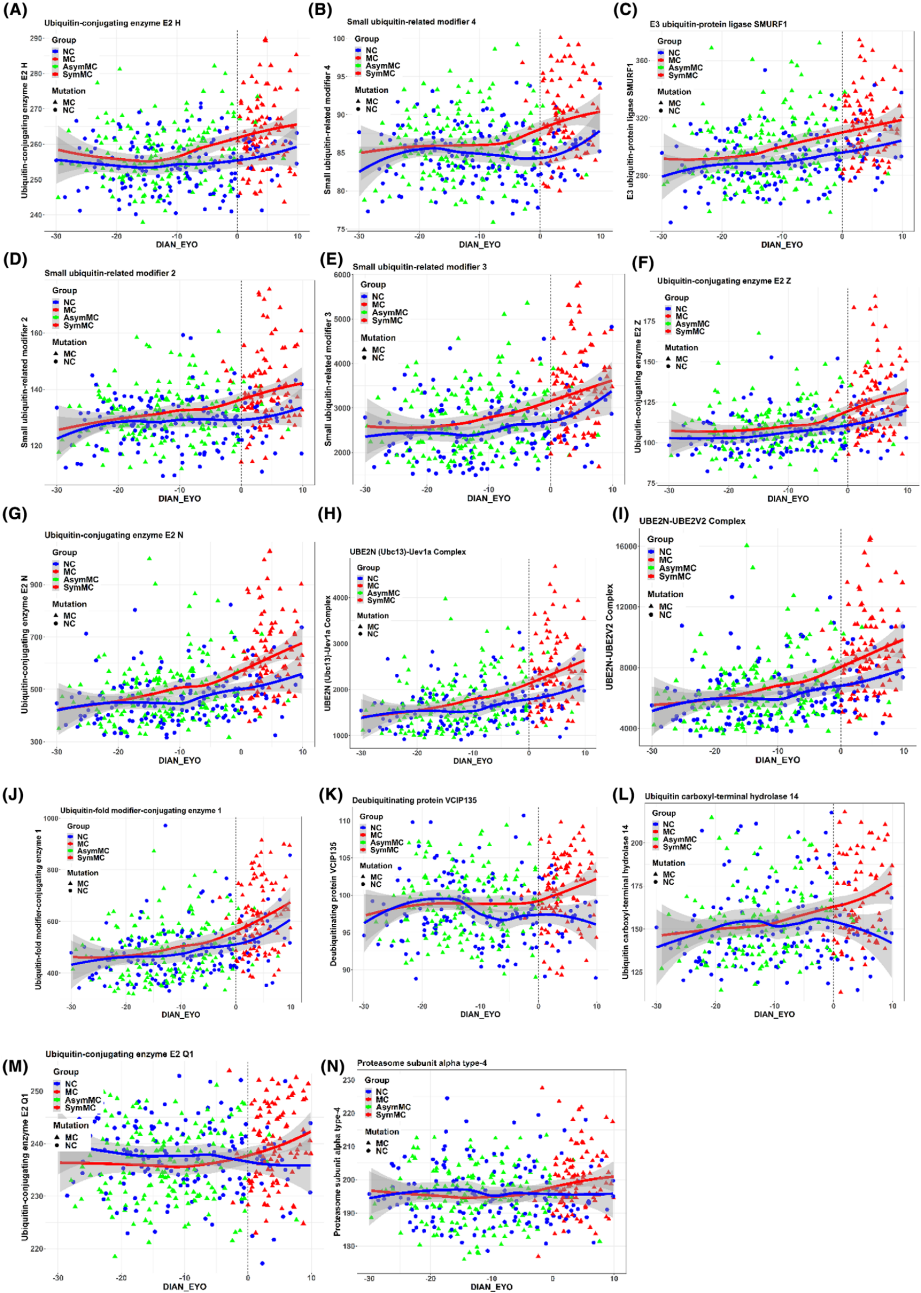
UPS protein levels relative to EYO at baseline: Depicted are asymptomatic mutation carriers, (green triangles, *n* = 179), symptomatic mutation carriers (red triangles, *n* = 104), and non‐carriers (blue circles, *n* = 172). Rows are organized by EYO when UPS protein levels began showing statistically significant elevation in mutation carriers (red line) compared to non‐carriers (blue line). The dotted line at 0 years indicates the anticipated symptom onset, with best‐fit curves represented by LOESS. A–C, E2H (–19 EYO each), SUMO4 (–18 EYO), and ubiquitin ligases: SMURF1 (–17 EYO). D‐F: E SUMO2, and SUMO3 (–16 EYO each). G–K, N, UBE2Z, UBE2N (–15 EYO each), UBE2N/Uev1a, UBE2N/UBE2V2 (–14 EYO each), UFC1 and deubiquinating enzyme VCIP135 (both –13 EYO). L–M, USP‐14 (–9 EYO), E2Q1, and PSMA4 close to EYO 0. Note: Parenthetical time points specify when proteins had significantly elevated levels in mutation carriers. DIAN, Dominantly Inherited Alzheimer Network; EYO, estimated years from symptom onset; MC, mutation carrier; NC, non‐carrier; UPS, ubiquitin–proteasome system.

Our LME model analysis identified a significant increase in CSF levels of 14 proteins when comparing MC to NC across EYO. These proteins encompassed six E2 enzymes (ubiquitin‐conjugating enzymes), one E3 enzyme (ubiquitin ligase), four ubiquitin modifiers, two deubiquitinases, and one proteasome component, all showing statistical significance with FDR *p* values < 0.05 (see Figure 1 and Table 2). Notably, in MCs, the cross‐sectional levels of certain proteins within the ubiquitin pathway began to elevate nearly two decades before the EYO. Specifically, between 15 and 20 years prior to the EYO, subtle increases were observed in proteins such as ubiquitin‐conjugating enzyme E2 H (UBE2H); the E3 ubiquitin ligase SMURF1 (SMURF1); and the small ubiquitin‐related modifiers 2, 3, and 4 (SUMO2, SUMO3, and SUMO4).[Fig alz70243-fig-0001], [Table alz70243-tbl-0002]

**TABLE 2 alz70243-tbl-0002:** 14 UPS protein list over DIAN EYO at baseline.

Protein_name	Slope estimate (MC vs. NC)	SE	Alpha	Lower 95% CI	Upper 95% CI	DIAN EYO^*^ Mutation *p* value
**E2 ubiquitin conjugating enzymes**						
Ubiquitin‐conjugating enzyme E2 H	0.22	0.07	0.05	0.09	0.36	0.0013
Ubiquitin‐conjugating enzyme E2 N	4.26	0.93	0.05	2.43	6.08	<0.0001
UBE2N (Ubc13)/Uev1a Complex	19.89	4.61	0.05	10.84	28.95	<0.0001
UBE2N/UBE2V2 Complex	73.11	17.57	0.05	38.59	107.64	<0.0001
Ubiquitin‐conjugating enzyme E2 Q1	0.21	0.06	0.05	0.10	0.32	0.0002
Ubiquitin‐conjugating enzyme E2 Z	0.48	0.13	0.05	0.23	0.72	0.0002
**E3 ubiquitin ligase **						
E3 ubiquitin‐protein ligase SMURF1	0.66	0.17	0.05	0.32	1.00	0.0001
**Ubiquitin modifiers**						
Small ubiquitin‐related modifier 2	0.31	0.08	0.05	0.14	0.47	0.0003
Small ubiquitin‐related modifier 3	19.62	6.17	0.05	7.50	31.75	0.0016
Small ubiquitin‐related modifier 4	0.11	0.04	0.05	0.04	0.18	0.0027
Ubiquitin‐fold modifier‐conjugating enzyme 1	3.27	0.91	0.05	1.47	5.06	0.0004
**Deubiquitinases **						
Ubiquitin carboxyl‐terminal hydrolase 14	0.60	0.19	0.05	0.24	0.97	0.0013
Deubiquitinating protein VCIP135	0.11	0.04	0.05	0.04	0.19	0.0017
**Proteasome components**						
Proteasome subunit alpha type‐4	0.23	0.07	0.05	0.08	0.37	0.0021

*Notes*: Individual slope per participant obtained from linear regression analyses representing the targeted UPS proteins of the study plotted against DIAN‐EYO. The β coefficients (slope estimates) for the linear and quadratic interaction terms in each regression model are shown in each panel (MC vs. NC). The standard errors (SE) are indicated in a separate column.

Abbreviations: CI, confidence interval; DIAN, Dominantly Inherited Alzheimer's Network; EYO, estimated years to symptom onset; MC, mutation carrier; NC, non‐mutation carriers; UPS, ubiquitin–proteasome system.

Between 10 and 15 years prior to symptom onset, multiple proteins within the UPS, particularly E2 ubiquitin‐conjugating enzymes, began to increase in MC compared to NC. These increases included ubiquitin‐conjugating enzyme E2 Z (UBE2Z), ubiquitin‐conjugating enzyme E2 N (UBE2N), the UBE2N/ubiquitin‐conjugating enzyme E2 variant 1A (Uev1a) complex, the UBE2N/ubiquitin‐conjugating enzyme E2 variant 2 (UBE2V2) complex, ubiquitin‐fold modifier‐conjugating enzyme 1 (UFC1), and the deubiquitinating protein VCIP135. In the decade leading up to and with symptom onset, a greater increase was observed in ubiquitin carboxyl‐terminal hydrolase 14 (USP‐14), ubiquitin‐conjugating enzyme E2 Q1 (UBE2Q1), and the proteasome subunit alpha type‐4 (PSMA4). The specific years showing significant differences are depicted in Figure 1. No modifying effects were observed based on sex, education level, or *APOE* ε4 status. Of note, nearly all these UPS proteins demonstrated the greatest difference between symptomatic MCs and NCs, with these differences diminishing in significance after excluding symptomatic MCs, suggesting a continuing rise with disease progression. See Table 2 and Table S1 in the supporting information.

### 3.3 Partial Spearman rank correlation analysis of UPS proteins and AD biomarkers

#### 3.3.1 Correlation analysis with amyloid‐related biomarkers and amyloid PET

**FIGURE 2:**
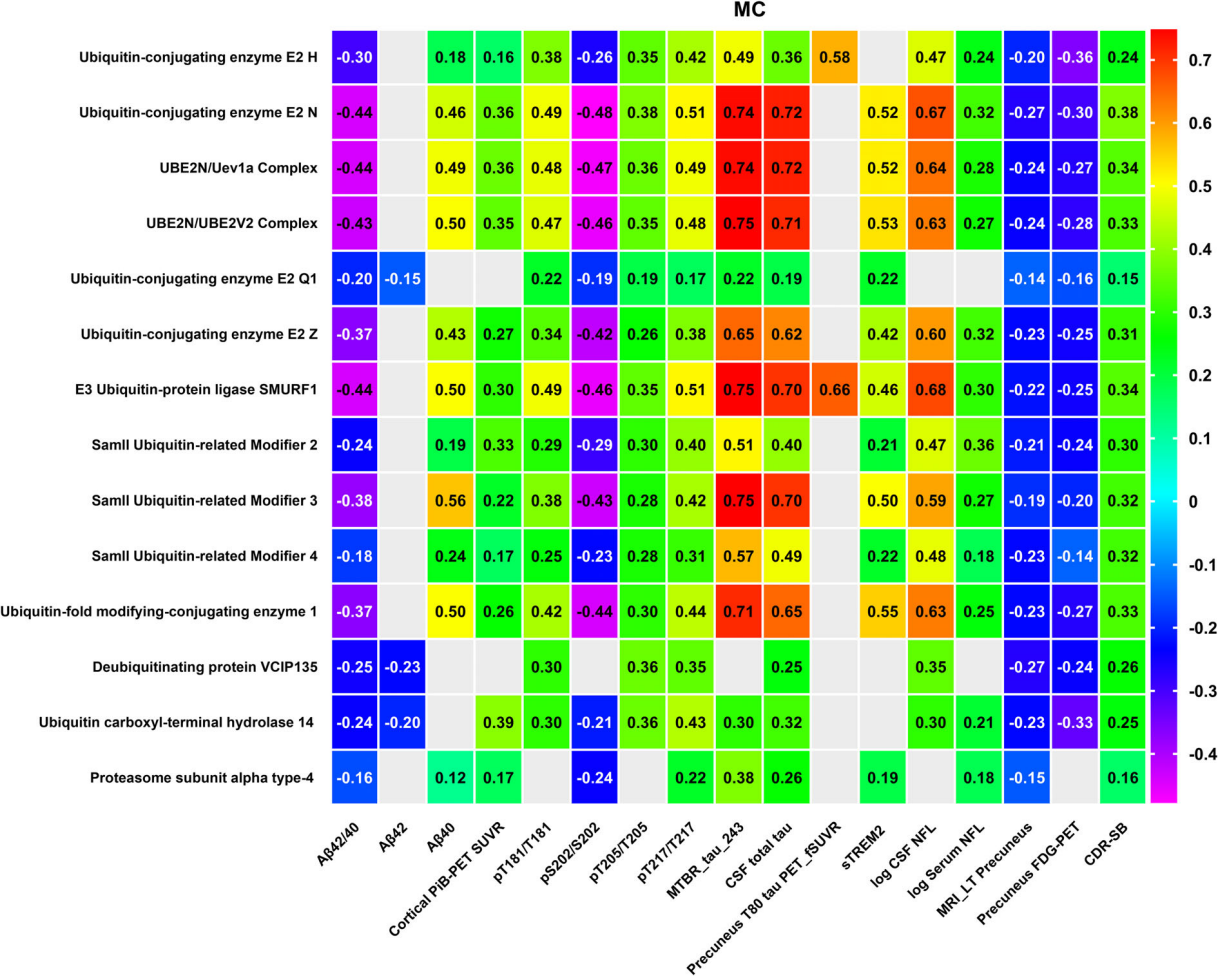
Heat map of Spearman correlations adjusted for age and sex between UPS proteins and neurological markers. This heatmap illustrates the results of a Spearman correlation analysis conducted between various UPS proteins and an array of AD biomarkers specific to the MC group. Each cell in the heatmap shows the Spearman correlation coefficient, with the scale indicated by the color gradient on the right: strong positive correlations are shown in red, strong negative correlations in purple, and no correlation in light blue. The UPS proteins are listed on the *y* axis, while the AD biomarkers are on the *x* axis, which includes amyloid and tau pathology markers, neurodegenerative markers from PET scans and MRI, as well as clinical assessment scales. The chart highlights statistically significant correlations (*r* values) across multiple dimensions, such as CSF Aβ42/40 ratio, Aβ40, Aβ42, cortical PiB PET SUVR, and various phosphorylated tau (pTau) ratios, alongside MTBR‐tau243, total tau (tTau), tau PET, CSF and serum NfL on a logarithmic scale, relative soluble TREM2 levels, MRI of the precuneus (left), and the Clinical Dementia Rating‐Sum of Boxes (CDR‐SB) in the MC group. All illustrated correlations are significant with a false discovery rate (FDR)–adjusted *p* value of < 0.05. Aβ, amyloid beta; AD, Alzheimer's disease; CSF, cerebrospinal fluid; FDG, fluorodeoxyglucose; MC, mutation carrier; MRI, magnetic resonance imaging; NfL, neurofilament light chain; PET, positron emission tomography; PiB, Pittsburgh compound B; SUVR, standardized uptake value ratio; TREM2, triggering receptor expressed on myeloid cells 2; UPS, ubiquitin–proteasome system.

After adjusting for age and sex, our analysis indicated that most of the 14 UPS proteins (see section 3.2) demonstrated mild to moderate correlations with cortical amyloid PET (PiB PET) SUVR in the MC group, in contrast to the NC group. The correlation coefficients varied from 0.16 to 0.39. Specifically, proteins such as UBE2N, UBE2N/Uev1a, UBE2N/UBE2V2, SMO2, E3 ubiquitin ligase SMURF1, and USP‐14 showed significant differences between the MC and NC groups (FDR *p* < 0.05). For more information, please see Figure 2 and Table 3.

**TABLE 3:** Comparison of protein level correlations with different biomarkers between mutation carriers (MC) and non‐carriers (NC).

Protein name	Correlation slope estimate (MC vs. NC)	SE	Alpha	Lower (95% CI)	Upper (95% CI)	*p* value (FDR *P*)
**CSF Aβ40**						
Ubiquitin‐fold modifier‐conjugating enzyme 1	−8.23	2.08	0.05	−12.31	−4.15	0.0003
Ubiquitin‐conjugating enzyme E2 Z	−65.47	17.12	0.05	−99.12	−31.82	0.0005
Ubiquitin‐conjugating enzyme E2 Q1	10.51	42.43	0.05	−72.88	93.90	0.8938
Ubiquitin‐conjugating enzyme E2 N	−12.59	2.23	0.05	−16.98	−8.20	<0.0001
Ubiquitin‐conjugating enzyme E2 H	−60.57	33.87	0.05	−127.13	5.99	0.1488
Ubiquitin carboxyl‐terminal hydrolase 14	13.19	12.91	0.05	−12.20	38.58	0.4534
UBE2N/UBE2V2 Complex	−0.52	0.12	0.05	−0.75	−0.29	<0.0001
UBE2N (Ubc13)/Uev1a Complex	−2.60	0.45	0.05	−‐3.48	−1.72	<0.0001
Small ubiquitin‐related modifier 4	−65.06	69.21	0.05	−201.08	70.96	0.4819
Small ubiquitin‐related modifier 3	−1.32	0.30	0.05	−1.91	−0.73	<0.0001
Small ubiquitin‐related modifier 2	−52.60	28.87	0.05	−109.34	4.14	0.1403
Proteasome subunit alpha type‐4	−1.75	30.65	0.05	−61.98	58.48	0.9804
E3 ubiquitin‐protein ligase SMURF1	−51.40	11.50	0.05	−74.00	−28.80	<0.0001
Deubiquitinating protein VCIP135	129.96	59.86	0.05	12.31	247.60	0.0677
**CSF Aβ42**						
Ubiquitin‐fold modifier‐conjugating enzyme 1	−1.97	0.25	0.05	−2.46	−1.48	<0.0001
Ubiquitin‐conjugating enzyme E2 Z	−13.69	2.01	0.05	−17.64	−9.74	<0.0001
Ubiquitin‐conjugating enzyme E2 Q1	−4.54	4.62	0.05	−13.63	4.54	0.4706
Ubiquitin‐conjugating enzyme E2 N	−2.39	0.26	0.05	−2.90	−1.87	<0.0001
Ubiquitin‐conjugating enzyme E2 H	−14.32	3.76	0.05	−21.71	−6.92	0.0005
Ubiquitin carboxyl‐terminal hydrolase 14	−2.60	1.41	0.05	−5.37	0.17	0.1358
UBE2N/UBE2V2 Complex	−0.11	0.01	0.05	−0.14	−0.09	<0.0001
UBE2N (Ubc13)/Uev1a Complex	−0.49	0.05	0.05	−0.59	−0.38	<0.0001
Small ubiquitin‐related modifier 4	−18.20	7.78	0.05	−33.49	−2.91	0.0453
Small ubiquitin‐related modifier 3	−0.29	0.04	0.05	−0.36	−0.21	<0.0001
Small ubiquitin‐related modifier 2	−11.65	3.20	0.05	−17.94	−5.35	0.0010
Proteasome subunit alpha type‐4	−5.16	3.39	0.05	−11.83	1.51	0.2229
E3 ubiquitin‐protein ligase SMURF1	−10.58	1.45	0.05	−13.42	−7.73	<0.0001
Deubiquitinating protein VCIP135	1.74	6.49	0.05	−11.02	14.51	0.8864
**CSF Aβ42/40**						
Ubiquitin‐conjugating enzyme E2 N	−0.000110	0.000021	0.05	0.000160	0.000070	<0.0001
Ubiquitin‐fold modifier‐conjugating enzyme 1	−0.000110	0.000021	0.05	0.000150	0.000070	<0.0001
Ubiquitin‐conjugating enzyme E2 Z	−0.000720	0.000157	0.05	0.001030	0.000410	<0.0001
Ubiquitin‐conjugating enzyme E2 Q1	−0.000600	0.000354	0.05	0.001300	0.000093	0.1686
Ubiquitin‐conjugating enzyme E2 H	−0.000880	0.000290	0.05	0.001450	0.000310	0.0066
Ubiquitin carboxyl‐terminal hydrolase 14	−0.000420	0.000105	0.05	0.000630	0.000220	0.0002
UBE2N/UBE2V2 Complex	−0.000006	0.000001	0.05	0.000008	0.000004	<0.0001
UBE2N (Ubc13)/Uev1a Complex	−0.000020	0.000004	0.05	0.000030	0.000010	<0.0001
Small ubiquitin‐related modifier 4	−0.000930	0.000597	0.05	0.002100	0.000244	0.2160
Small ubiquitin‐related modifier 3	−0.000010	0.000003	0.05	0.000020	0.000007	0.0001
Small ubiquitin‐related modifier 2	−0.000640	0.000246	0.05	0.001120	0.000150	0.0239
Proteasome subunit alpha type‐4	−0.000470	0.000266	0.05	0.000990	0.000057	0.1570
E3 ubiquitin‐protein ligase SMURF1	−0.000520	0.000111	0.05	0.000740	0.000300	<0.0001
Deubiquitinating protein VCIP135	−0.001150	0.000523	0.05	0.002180	0.000120	0.0648
**CSF total tau**						
Ubiquitin‐fold modifier‐conjugating enzyme 1	1.51	0.21	0.05	1.09	1.93	<0.0001
Ubiquitin‐conjugating enzyme E2 Z	9.89	1.69	0.05	6.56	13.22	<0.0001
Ubiquitin‐conjugating enzyme E2 Q1	6.38	4.34	0.05	−2.16	14.92	0.2402
Ubiquitin‐conjugating enzyme E2 N	1.41	0.23	0.05	0.95	1.86	<0.0001
Ubiquitin‐conjugating enzyme E2 H	15.40	3.35	0.05	8.81	21.98	<0.0001
Ubiquitin carboxyl‐terminal hydrolase 14	5.72	1.25	0.05	3.26	8.19	<0.0001
UBE2N/UBE2V2 Complex	0.08	0.01	0.05	0.05	0.10	<0.0001
UBE2N (Ubc13)/Uev1a Complex	0.28	0.05	0.05	0.19	0.38	<0.0001
Small ubiquitin‐related modifier 4	28.50	6.25	0.05	16.21	40.80	<0.0001
Small ubiquitin‐related modifier 3	0.24	0.03	0.05	0.18	0.30	<0.0001
Small ubiquitin‐related modifier 2	10.65	2.89	0.05	4.96	16.33	0.0009
Proteasome subunit alpha type‐4	10.30	3.23	0.05	3.94	16.65	0.0044
E3 ubiquitin‐protein ligase SMURF1	7.02	1.13	0.05	4.80	9.24	<0.0001
Deubiquitinating protein VCIP135	21.79	6.46	0.05	9.08	34.50	0.0025
**CSF pS202/S202**						
Ubiquitin‐fold modifier‐conjugating enzyme 1	−0.00096	0.000802	0.05	−0.00254	0.00062	0.36
Ubiquitin‐conjugating enzyme E2 Z	−0.00572	0.006346	0.05	−0.01821	0.006781	0.51
Ubiquitin‐conjugating enzyme E2 Q1	−0.00568	0.01469	0.05	−0.03461	0.02325	0.84
Ubiquitin‐conjugating enzyme E2 N	−0.00017	0.000962	0.05	−0.00207	0.00172	0.92
Ubiquitin‐conjugating enzyme E2 H	−0.00836	0.01203	0.05	−0.03206	0.01533	0.63
Ubiquitin carboxyl‐terminal hydrolase 14	−0.0082	0.004665	0.05	−0.0174	0.000991	0.16
UBE2N/UBE2V2 Complex	−0.00001	0.000049	0.05	−0.00011	0.000086	0.90
UBE2N (Ubc13)/Uev1a Complex	−7.24E‐06	0.000185	0.05	−0.00037	0.000357	0.98
Small ubiquitin‐related modifier 4	−0.04379	0.02524	0.05	−0.09349	0.005914	0.16
Small ubiquitin‐related modifier 3	−0.00003	0.000125	0.05	−0.00028	0.000212	0.89
Small ubiquitin‐related modifier 2	0.002304	0.01054	0.05	−0.01845	0.02305	0.90
Proteasome subunit alpha type‐4	−0.01055	0.01091	0.05	−0.03205	0.01094	0.47
E3 ubiquitin‐protein ligase SMURF1	−0.00372	0.004526	0.05	−0.01263	0.005195	0.55
Deubiquitinating protein VCIP135	−0.03129	0.02204	0.05	−0.0747	0.01212	0.26
**CSF MTBR‐tau243**						
Ubiquitin‐fold modifier‐conjugating enzyme 1	0.002096	0.000433	0.05	0.00124	0.002951	<0.0001
Ubiquitin‐conjugating enzyme E2 Z	0.01411	0.003422	0.05	0.007353	0.02086	0.0002
Ubiquitin‐conjugating enzyme E2 Q1	0.0132	0.008518	0.05	−0.00361	0.03001	0.2165
Ubiquitin‐conjugating enzyme E2 N	0.001955	0.000394	0.05	0.001176	0.002734	<0.0001
Ubiquitin‐conjugating enzyme E2 H	0.02597	0.006701	0.05	0.01274	0.03919	0.0005
Ubiquitin carboxyl‐terminal hydrolase 14	0.006676	0.002117	0.05	0.002495	0.01086	0.0053
UBE2N/UBE2V2 Complex	0.000097	0.000022	0.05	0.000053	0.00014	<0.0001
UBE2N (Ubc13)/Uev1a Complex	0.00037	0.000085	0.05	0.000202	0.000538	0.0001
Small ubiquitin‐related modifier 4	0.03977	0.01258	0.05	0.01494	0.0646	0.0052
Small ubiquitin‐related modifier 3	0.000257	0.000066	0.05	0.000127	0.000387	0.0005
Small ubiquitin‐related modifier 2	0.01727	0.005147	0.05	0.007114	0.02743	0.0028
Proteasome subunit alpha type‐4	0.01882	0.006232	0.05	0.006523	0.03112	0.0073
E3 ubiquitin‐protein ligase SMURF1	0.007931	0.002276	0.05	0.003439	0.01242	0.0019
Deubiquitinating protein VCIP135	0.03418	0.01332	0.05	0.007879	0.06047	0.0260
**CSF log NfL**						
Ubiquitin‐fold modifier‐conjugating enzyme 1	0.001010	0.000953	0.05	0.000880	0.002903	0.44
Ubiquitin‐conjugating enzyme E2 Z	0.008744	0.007894	0.05	0.006930	0.024420	0.41
Ubiquitin‐conjugating enzyme E2 Q1	−0.002900	0.016150	0.05	0.034980	0.029190	0.92
Ubiquitin‐conjugating enzyme E2 N	0.000904	0.001149	0.05	0.001380	0.003185	0.57
Ubiquitin‐conjugating enzyme E2 H	0.019230	0.014370	0.05	0.009320	0.047770	0.30
Ubiquitin carboxyl‐terminal hydrolase 14	0.006174	0.004911	0.05	0.003600	0.015950	0.34
UBE2N/UBE2V2 Complex	0.000035	0.000060	0.05	0.000080	0.000154	0.71
UBE2N (Ubc13)/Uev1a Complex	0.000099	0.000222	0.05	0.000340	0.000539	0.80
Small ubiquitin‐related modifier 4	0.050810	0.027140	0.05	0.003100	0.104700	0.13
Small ubiquitin‐related modifier 3	0.000256	0.000157	0.05	0.000060	0.000568	0.20
Small ubiquitin‐related modifier 2	0.020350	0.010450	0.05	0.000410	0.041110	0.12
Proteasome subunit alpha type‐4	0.019370	0.011950	0.05	0.004370	0.043100	0.20
E3 ubiquitin‐protein ligase SMURF1	0.008392	0.005402	0.05	0.002340	0.019120	0.22
Deubiquitinating protein VCIP135	0.045400	0.022490	0.05	0.000736	0.090070	0.10
**CSF sTREM2**						
Ubiquitin‐fold modifier‐conjugating enzyme 1	−0.001090	0.001530	0.05	0.004110	0.001930	0.62
Ubiquitin‐conjugating enzyme E2 Z	−0.005930	0.012730	0.05	0.031040	0.019170	0.79
Ubiquitin‐conjugating enzyme E2 Q1	0.033380	0.030710	0.05	0.027210	0.093970	0.42
Ubiquitin‐conjugating enzyme E2 N	−0.002500	0.001694	0.05	0.005840	0.000845	0.24
Ubiquitin‐conjugating enzyme E2 H	0.010570	0.025510	0.05	0.039750	0.060880	0.82
Ubiquitin carboxyl‐terminal hydrolase 14	−0.002600	0.009801	0.05	0.021960	0.016760	0.89
UBE2N/UBE2V2 Complex	−0.000120	0.000089	0.05	0.000300	0.000053	0.28
UBE2N (Ubc13)/Uev1a Complex	−0.000620	0.000331	0.05	0.001270	0.000032	0.13
Small ubiquitin‐related modifier 4	−0.037690	0.048930	0.05	0.134200	0.058850	0.58
Small ubiquitin‐related modifier 3	−0.000200	0.000242	0.05	0.000680	0.000274	0.54
Small ubiquitin‐related modifier 2	0.007498	0.021480	0.05	0.034880	0.049870	0.86
Proteasome subunit alpha type‐4	0.022260	0.022370	0.05	0.021870	0.066400	0.47
E3 ubiquitin‐protein ligase SMURF1	−0.000200	0.009243	0.05	0.018430	0.018040	0.99
Deubiquitinating protein VCIP135	−0.003390	0.049390	0.05	0.100800	0.094050	0.98

*Notes*: Individual slope comparison with different biomarkers was obtained from linear regression analyses, representing the targeted UPS proteins of the study plotted against DIAN‐EYO. The β coefficients (slope estimates) for the linear interaction terms in each regression model are shown for each panel (MC vs. NC), with their corresponding standard errors (SE) detailed in a separate column. The 95% CI for each slope estimate is split into two parts: the lower 95% CI indicates the lower bound, and the upper 95% CI indicates the upper bound of the interval. A *p* value < 0.05 is generally considered to indicate statistical significance.

Abbreviations: Aβ, amyloid beta; CI, confidence interval; CSF, cerebrospinal fluid; DIAN, Dominantly Inherited Alzheimer's Network; EYO, estimated years to symptom onset; FDR, false discovery rate; MC, mutation carrier; MRI, magnetic resonance imaging; NC, non‐mutation carrier; NfL, neurofilament light chain; sTREM2, soluble triggering receptor expressed on myeloid cells 2; t‐tau, total tau.

The correlations of the 14 UPS proteins with soluble CSF Aβ were significantly inversely related to the Aβ42/40 ratio in the MC group, with *r* values ranging from –0.16 to –0.44. This pattern is largely attributed to a positive association with Aβ40. While NCs also demonstrated several associations with soluble Aβ, proteins such as UBE2N, UBE2N/Uev1a, UBE2N/UBE2V2, and UFC1 positively correlated with Aβ42. In contrast, the associations with Aβ40 were more pronounced in NCs compared to MCs. For additional information, please refer to Figure 2 and Table 3.

#### 3.3.2 Correlation with tau‐related biomarkers

We also evaluated the associations between those 14 UPS proteins and flortaucipir uptake in the precuneus for both MC and NC, using Spearman correlation models adjusted for age and sex. Proteins including UBE2H and E3 ubiquitin‐protein ligase SMURF1 demonstrated moderate to strong associations with elevated tau PET signal in the precuneus, with *r* values ranging from 0.58 to 0.66 in the MC group (*p* < 0.05, FDR 5%). No significant association was observed in the NC group; see Figure 2 and Table 3.

We identified significant correlations between CSF total tau in both the MC and NC groups, with each group showing a substantial correlation. These proteins including UBE2N, UBE2N (Ubc13)/Uev1a Complex, UBE2N/UBE2V2 Complex, UBE2H, UBE2Q1, UBE2Z, E3 ubiquitin‐protein ligase SMURF1, SUMO2, SUMO3, SUMO4, UFC1, USP14, Deubiquitinating protein VCIP‐135 andPSMA4, exhibited stronger correlation coefficients in MC group, ranging from ≈ 0.25 to 0.72 (*p* < 0.05, FDR 5%; see Figure 2 and Table 3 for details).

In the MC group, all of the aforementioned 14 UPS proteins, with the exception of PSMA4, exhibited a positive correlation with pTau181/T181, with *r* values ranging from 0.22 to 0.49, and with pTau205/T205, for which *r* values ranged from 0.19 to 0.38. Furthermore, these 14 UPS proteins also showed a positive correlation with pTau217/T217, with *r* values spanning from 0.17 to 0.51. Conversely, 13 out of the 14 UPS proteins, excluding the deubiquitinating protein VCIP‐135, demonstrated a negative correlation with pS202/S202, with correlation coefficients ranging from ≈ –0.21 to –0.48.

The strongest correlations were observed with 13 of these 14 proteins, excluding the deubiquitinating protein VCIP‐135 and MTBR‐tau243, in both MC and NC groups. Their *r* values varied from ≈ 0.3 to 0.75 in the MC group. Although statistically significant correlations were identified in NCs, those in MCs were 3.3 ‐10 times greater based on the model estimated correlations, the β coefficients ranging from 0.000098 to 0.02581 (MC vs. NC). Notably, the deubiquitinating protein VCIP‐135 was negatively associated with MTBR‐tau243, with an *r* value of –0.41 in the NC group. Table 3 outlines the absolute differences in beta coefficients between MC and NC groups. For further details, refer to Figure 2 (MC) and Table 3 (MC vs. NC).

#### 3.3.3 Correlation analysis with neurodegeneration and clinical state

To investigate the relationship between CSF UPS protein levels and imaging markers of neurodegeneration, as well as clinical stages, we conducted correlation analyses with various imaging parameters. These included FDG composite and MRI‐based precuneus cortical thickness, alongside the CDR‐SB. Acknowledging the established correlation between age, sex, AD disease stage, and the age‐associated increase in numerous proteostasis peptides, we adjusted the correlations for both age and sex.^32^, ^37^, ^38^ Our findings reveal that all 14 UPS proteins exhibited a significant, though mild to moderate, positive correlation with CDR‐SB in the MC (see Figure 2). Moreover, most UPS proteins—except UBE2H—demonstrated a significant negative association with cognitive performance, as measured by a composite of global cognition (Mini‐Mental State Examination [MMSE]), verbal immediate recall (MEMUNITS), Wechsler Adult Intelligence Scale Digit Symbol Substitution Test (WAIS‐DSST), and delayed verbal recall (WORDDEL). These associations were observed after adjusting for sex and *APOE* status, using a cognitive composite score computed as a linear combination of the four psychometric measures. Detailed results are presented in Figure S1 and Table S2 in supporting information.

All UPS proteins, except PSMA4, demonstrated mild to moderate negative correlations with FDG PET in the precuneus region for the MC group, with *r* values ranging from –0.14 to –0.36. Additionally, all 14 UPS proteins displayed a mild negative correlation with MRI findings in the precuneus region (left), with *r* values ranging from –0.14 to –0.27.

#### 3.3.4 Correlation with CSF and serum NfL

We identified a significant positive correlation between the logarithmic values (log) of CSF and serum NfL. Notably, within the MC impairment group, CSF NfL demonstrated moderate to high positive correlations with 12 of the 14 UPS proteins discussed in section 3.2, excluding UBE2Q1 and PSMA4. The correlation coefficients (*r*) ranged from 0.3 to 0.68. In the NC group, UBE2N, UBE2N/Uev1a, UBE2N/UBE2V2, UBE2Z, and UFC1 also showed positive correlations with CSF NfL, albeit the associations were more marked in the MC group compared to the NC group. Furthermore, 12 out of these 14 proteins, with the exception of UBE2Q1 and the deubiquitinating protein VCIP‐135, exhibited mild to moderate positive correlations with serum NfL in the MC group, with correlation coefficients ranging from ≈ 0.18 to 0.36.

#### 3.3.5 Correlation with sTREM2

We observed a significant positive correlation between the normalized levels of CSF sTREM2 (normalized using an internal standard, termed relative sTREM2) and a selection of 14 UPS proteins in both MC and NC groups. In the MC group, all proteins except UBE2H, USP‐14, and VCIP‐135 showed positive correlations with sTREM2, with *r* values ranging from ≈ 0.21 to 0.55. In the NC group, proteins such as UBE2N, the UBE2N/Uev1a Complex, the UBE2N/UBE2V2 Complex, UBE2Z, E3 ubiquitin‐protein ligase SMURF1, SUMO3, and UFC1 also displayed positive correlations with sTREM2, with *r* values ranging from 0.43 to 0.56, which reached statistical significance (*p* < 0.05, FDR 5%). However, Figure 1 illustrates that NCs maintain very normal levels for nearly all these 14 proteins, suggesting that the observed correlations might be driven by a much smaller variance rather than a greater range. Furthermore, the model‐estimated correlations analysis in Table 3 indicated that the β coefficients showed no significant difference between MC and NC for all 14 UPS proteins. This observation suggests that the associations may not necessarily reflect biological phenomena but could instead be attributed to characteristics of the assay.

### 3.4 UPS protein levels across biological stages

**FIGURE 3:**
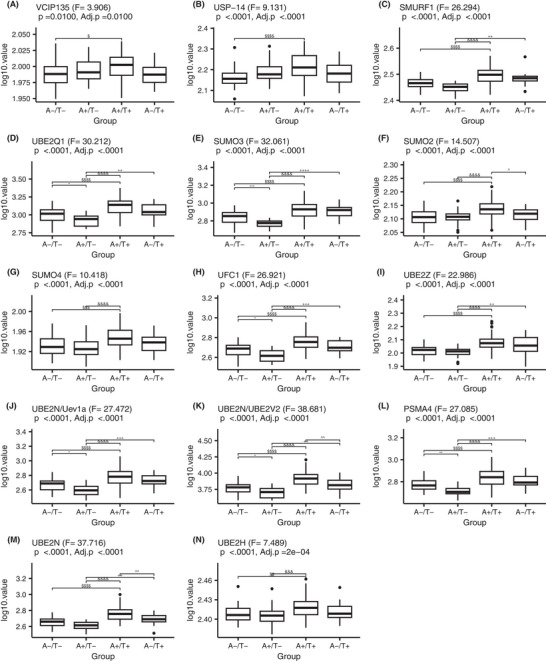
UPS protein levels across A/T biological stages. The box plots illustrate the log₁₀‐transformed protein levels of 14 UPS proteins across four amyloid PET/total tau (A/T) classification groups: A–/T–, A+/T–, A+/T+, and A–/T+. The following proteins are shown: (A) VCIP135, (B) USP‐14, (C) SMURF1, (D) UBE2Q1, (E) SUMO3, (F) SUMO2, (G) SUMO4, (H) UFC1, (I) UBE2Z, (J) UBE2N/Uev1a, (K) UBE2N/UBE2V2, (L) PSMA4, (M) UBE2N, (N) UBE2H. The *x* axis represents the A/T groups, while the *y* axis denotes the log₁₀‐transformed protein levels. Statistical significance was assessed using analysis of variance for overall differences, followed by Tukey's honestly significant difference test for pairwise comparisons. The *p* values for each protein are indicated in each plot, with significant pairwise differences marked by different symbols, representing different levels of significance. A represents amyloid PET, T refers to total tau. Statistical significance is denoted as follows: *, +, ^, &, $ for *p* < 0.05; **, ^^, ++, &&, $$ for *p* < 0.001; ***, +++, ^^^, &&&, $$$ for *p* < 0.000; and ****, ++++, ^^^^, &&&&, for *p* < 0.00001. PET, positron emission tomography; UPS, ubiquitin–proteasome system.

UPS protein levels were analyzed across biological stages, with amyloid (A) and tau (T) classification determined using Lumipulse total tau at a cutoff of 270.5, selected based on its predictive accuracy for amyloid PET positivity among mutation carriers. Amyloid positivity was defined as an amyloid burden of SUVR ≥ 1.25.^39^ To account for the wide range of protein expression levels, log₁₀ transformation was applied for normalization and comparison. The distribution of each protein across the A–/T–, A+/T–, A+/T+, and A–/T+ groups was visualized using box plots, with ANOVA used to assess overall differences and Tukey's honestly significant difference test for pairwise comparisons. After FDR adjustment, all 14 UPS proteins exhibited a gradual increase from A–/T– to A+/T+, with a mild decrease in UFC1, UBE2N/Uev1a, UBE2N/UBE2V2, and PSMA4 at the early stage (A–/T– to A+/T–), indicating progressive dynamic changes in UPS activity in relation to amyloid and tau pathology; see Figure 3. Based on the recently updated Alzheimer's Association AD Diagnostic Criteria,^40^ we repeated the same analysis using a Core 2 (late tau) biomarker, CSF pT205/T205 (The cutoff value is 0.479). We found similar patterns in which nearly all UPS‐related proteins increased from A–/T– to A+/T+. The exception was that there were fewer statistically significant differences identified in the A–/T+ group (non‐AD pattern) than when using the total tau immunoassay; see Figure S2 in supporting information.

## 4 DISCUSSION

In our study, we observed that levels of 14 UPS proteins were elevated in the DIAD MC group across different stages of the disease. Notably, these differences increased closer to the time of predicted clinical symptom onset, continued to rise with symptom progression (Figure 1), and were unaffected by specific gene mutation. Further, our findings reveal consistently stronger associations with MTBR‐tau243, total tau, tau PET, and CSF NfL, suggesting a more specific link with tau aggregation and neurodegeneration. Yet, the additional, albeit weaker, correlations between rising levels of UPS‐related proteins and markers of neurodegeneration, phosphorylated tau (ptauT181/T181, ptauS202/S202, ptauT205/T205, ptauT217/T217), and the Aβ 42/40 ratio provides support for activation of the UPS and proteostasis/autophagy pathway earlier in the asymptomatic stage of DIAD.

### 4.1 Proteasome and AD

The UPS, with the proteasome as its essential component, is crucial for degrading ubiquitinated proteins. The proteasome is a barrel‐shaped 20S complex composed of four types of subunits (α, β, γ, δ), with the β subunits having peptide‐cleaving capabilities.^41^ Protein oxidation and excessive phosphorylation could impede the proteasome's key roles in intracellular protein quality control and the processing of Aβ and tau, potentially influencing AD pathology.^42^ The proteasome's role in AD remains underexplored. Our study uniquely contributes to the literature by assessing multiple proteins within the proteasome system across the disease spectrum and provides multiple opportunities for further exploration.

The correlations between UPS proteins and tau markers in DIAD highlight a complex relationship among protein degradation, tau pathology, and amyloid accumulation. Stronger associations with tau biomarkers suggest that UPS dysfunction or compensatory upregulation may be more closely tied to tau aggregation than directly to Aβ plaques, as UPS protein levels were generally similar between NCs and MCs during early amyloid pathology. Notably, these correlations were strongest with late‐stage tau markers,^40^ raising questions about the timing and mechanisms linking amyloid pathology, tau accumulation, and UPS activity in DIAD. This suggests that while amyloid mutations may initiate the disease, neurodegeneration severity may be more directly tied to tau pathology and its interaction with protein degradation.^43^ The differential UPS correlations with various phosphorylated tau species might reflect specific UPS responses to distinct tau pathologies.^44^ These insights emphasize the importance of tau‐targeted therapies and UPS modulation as potential strategies, particularly in genetically at‐risk populations such as DIAD, in which early‐onset mutations accelerate the pathological cascade. Importantly, the stronger association of UPS proteins with tau‐related biomarkers suggests that although DIAD mutations alter Aβ processing resulting in early Aβ pathology, it is not until there is a substantial tau pathological burden that abnormalities in the UPS are identified. This sequence could further support the role of Aβ pathology driving the development of intracellular tau tangles, but possibly through an eventual decompensation of UPS‐related proteostasis. Whether this is unique to DIAD will require similar analyses in late‐onset AD.

### 4.2 UPS, autophagy, and AD

**FIGURE 4:**
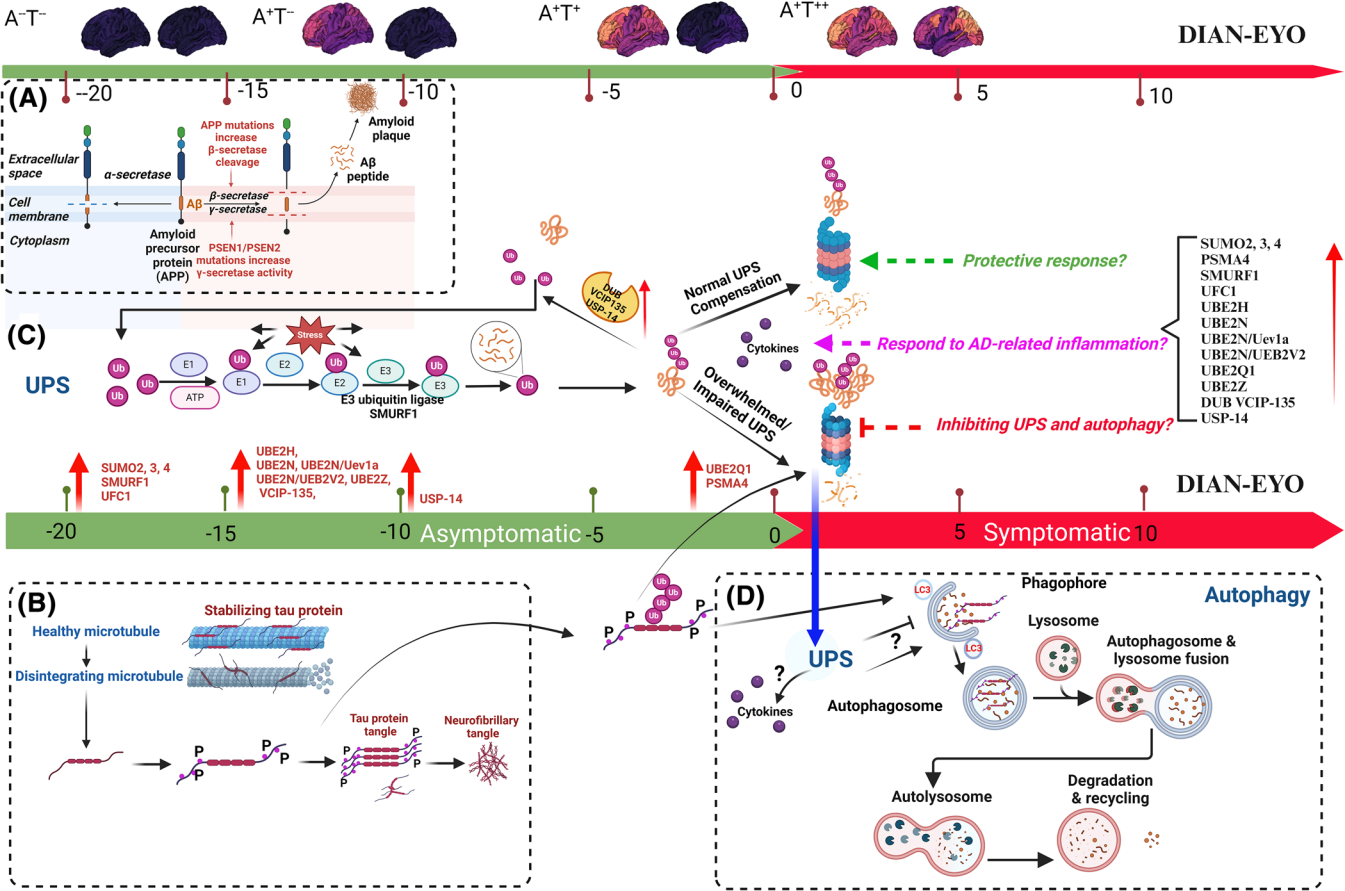
Pathophysiological progression of UPS in DIAD. This figure depicts the temporal progression of UPS alterations in DIAD from 20 years before symptom onset (EYO –20) to 10 years after (EYO 10). It contrasts the homeostatic and dysregulated states of the UPS and autophagy pathways, as illustrated in panels C and D. The schema underscores the pathological evolution from amyloid beta accumulation into plaques (A) to the aggregation of tau protein into neurofibrillary tangles (B). Early ubiquitination with neuritic plaque development may represent an adaptive response to tau phosphorylation/plaque development (EYO –20 to –10; C); however, with disease progression and the development of more widespread intraneuronal NFTs, the UPS system may become overwhelmed or impaired, precipitating a compensatory increase in autophagic activity. This hypothesis posits that the interplay between UPS and autophagy reflects a dynamic cellular attempt to mitigate the escalating tauopathy and amyloid plaques, with an initial robust UPS reaction potentially yielding to a heightened autophagic response as the disease progresses. Created with BioRender.com. The DIAN‐EYO timeline is denoted in years. A^−^, amyloid negative; A^+^, amyloid positive; DIAD, Dominantly inherited Alzheimer's disease; DIAN, Dominantly Inherited Alzheimer's Network; EYO, estimated years from symptom onset; NFT, neurofibrillary tangle; T^−^, tau negative; T^+^, tau positive; UPS, ubiquitin–proteasome system.

The UPS and autophagy are key protein degradation pathways in eukaryotes, each targeting different types of substrates: the UPS primarily manages short‐lived, misfolded soluble proteins, while autophagy handles longer‐lived proteins, insoluble aggregates, and organelles. Both systems rely on ubiquitin for target recognition, emphasizing their role in cellular health and proteostasis.^45^ The E3 ubiquitin‐protein ligase SMURF1 is particularly vital in autophagy regulation by activating PPP3/calcineurin and transcription factor EB (TFEB), underscoring the lysosome's role in cell signaling. SMURF1 also influences lysosomal biogenesis and, in concert with PPP3/calcineurin, supports the autolysosome pathway, indirectly aiding autophagosome maturation via TFEB regulation.^46^ In our study, SMURF1 upregulation suggests it may function as a protective mechanism enhancing protein quality control or might contribute to AD pathogenesis through autophagy regulation. SMURF1's role in DIAD and its interactions with autophagy in AD warrant further investigation to clarify their impact on disease progression. The UPS's role in DIAD and potential mechanisms are illustrated in Figure 4.

Our findings reveal significantly elevated levels of MAPLC3A and MAPLC3B in the MC group compared to the NC group across DIAN‐EYO (Figure S3A and B in supporting information). These proteins, essential for autophagosome formation, may indicate autophagy involvement in DIAD.^47^ While changes in SMURF1 might suggest autophagy dysregulation, the concurrent MAPLC3 alterations strengthen this association. The lack of significant changes in other core autophagy proteins (e.g., LAMP1, P62) does not exclude autophagy involvement, as autophagy is a multi‐step process in which protein levels may vary independently of autophagy flux.^48^ This complexity highlights the need for future studies focusing on autophagy flux and component interactions to better understand UPS and autophagy dysfunction in DIAD and its potential as a therapeutic target.^49^

### 4.3 The role of E2, E3 enzymes in AD and other neurodegenerative diseases

Alterations in the Ube2 subfamily genes, notably *UBE2N*, play a significant role in AD and other neurodegenerative disorders.^50^, ^51^ Changes in the expression and methylation of *UBE2N* and its complexes suggest their involvement in AD pathologies, such as protein aggregation and genomic regulation.^52^, ^53^ Recent research using Gene Expression Omnibus (GEO) data identified *UBE2N* as an immune‐related biomarker for AD,^50^ linked to T cell and B cell functions and synaptic signaling.^54^ Suppressing UBE2N has been shown to alleviate AD pathology by enhancing Aβ clearance in mouse models, marking it as a potential therapeutic target.^51^ Moreover, heterodimers like UBE2V1‐a, involved in atypical polyubiquitination, impacting inflammation and proteasomal degradation.^55^ Of note, the Ube2 proteins were shown to have high correlations with sTREM2, potentially linking the elevation of this group of UPS pathways with inflammation and protein aggregation. Other UBE2 enzymes, such as UBE2I, UBE2Q1, UBE2E1, and UBE2Z, display varied regulatory patterns in neurodegenerative diseases like frontotemporal dementia, suggesting the Ube2 family's extensive influence on neurodegeneration, inflammation, and cellular stress responses.^50^, ^51^, ^55^

Our study highlights significant changes in E3 ubiquitin ligases, especially SMURF1, which is associated with aggresome formation in AD, a mechanism to prevent the toxic spread of misfolded proteins.^56^, ^57^ SMURF1's localization in Hirano bodies^57^ may be one explanation for the elevation seen as protein aggregates accumulate with disease progression. Relatedly, it is unclear if the increased levels of this group of UPS proteins and their correlation with neurodegeneration markers and tau biomarkers are just a reflection of late‐stage protein aggregation, or if this pathway contributes to the development of neurodegeneration.^56^, ^58^, ^59^ This underlines the need to further explore the Ube2 family and SMURF1's roles in AD progression and their therapeutic possibilities.

### 4.4 Ubiquitin modifiers and AD

UFC1 is significantly associated with AD, playing a crucial role in protein folding, secretion, and endoplasmic reticulum (ER) stress, and our study supports this finding, showing a strong positive correlation between UFC1 and CSF NfL and a moderate correlation with total tau, a marker of later stages of disease.^60^, ^61^ Additionally, our study enhances understanding of post‐translational modifications in AD through the role of SUMOs and SUMOylation.^59^, ^62^, ^63^ We observed increased levels of SUMO2, SUMO3, and SUMO4 in individuals with DIAD mutations, implicating SUMOylation in AD pathophysiology. Moreover, SUMOylation's involvement in tau phosphorylation suggests its impact on tau stability and degradation, contributing to AD's characteristic NFTs and neuronal loss.^59^, ^63^

### 4.5 Deubiquitinase and AD

In AD, alterations in DUBs underscore their critical role in maintaining ubiquitination balance and their potential involvement in disease progression.^64^, ^65^ Our study identified elevated levels of deubiquitinating protein VCIP‐135 and USP‐14, which are involved in cellular homeostasis and protein processing, within the group. This elevation might suggest a compensatory mechanism in response to the misfolded proteins characteristic of AD or a role in the proteasomal degradation process.^65^, ^66^, ^67^ However, our findings did not reveal a significant role for UCHL‐1 in AD progression, indicating that its involvement may vary across different stages of the disease.

In conclusion, our study reveals early and dynamic changes in UPS proteins that correlate with established AD biomarkers. While these changes do not supersede established AD biomarkers, they offer a complementary perspective on AD pathogenesis, highlighting the importance of protein quality control systems in the disease process. By integrating UPS dysfunction into our understanding of AD, we open new avenues for biomarker development and therapeutic intervention. Future research building on these findings has the potential to significantly advance our understanding of AD mechanisms and improve strategies for the treatment of this devastating disease. These findings necessitate further research to explore these proteins’ roles in misfolded protein aggregation and their impact on other degradation systems like autophagy.^48^, ^49^

## CONFLICT OF INTEREST STATEMENT

RJB is the director of the DIAN‐TU and principal investigator of DIAN and the DIAN‐TU‐001 trial. Unrelated to this study, for the DIAN‐TU, he receives research support from the NIH, Eli Lilly and Company, F. Hoffman‐La Roche, Ltd., Eisai, Alzheimer's Association, GHR Foundation, Anonymous Organization, DIAN‐TU Pharma Consortium (Active Members: Biogen, Eisai, Eli Lilly and Company, Janssen, F. Hoffmann‐La Roche, Ltd./Genentech). JH is a paid consultant for F. Hoffmann‐La Roche, Ltd., Prothena, and Parabon Nanolabs, and is on a data safety and monitoring board (DSMB) for Eisai. EMM receives grant funding from NIA; Institutional funding from Eli Lilly, Hoffmann‐La Roche, Eisai. He is a DSMB member (paid directly) for Alector; Eli Lilly; a scientific advisory board member (paid directly to him) for Alzamend, Fondation Alzheimer. He acts as a consultant/advisor for Sage Therapeutics, Eli Lilly, Sanofi, AstraZeneca, Hoffmann La‐Roche. CC has received research support from GSK and EISAI. The funders of the study had no role in the collection, analysis, or interpretation of data; in the writing of the report; or in the decision to submit the paper for publication. CC is a member of the advisory board of Circular Genomics and owns stocks in these companies. DP is an employee of GlaxoSmithKline (GSK) and holds stock in GSK. CX is supported by National Institute on Aging (NIA) grants R01 AG067505 and R01 AG053550. JCM is the Friedman Distinguished Professor of Neurology, Associate Director, Knight ADRC; Associate Director of DIAN, and Founding Principal Investigator of DIAN. He is funded by NIH grants # P30 AG066444; P01AG003991, P01AG026276, and U19 AG024904. Neither he nor his family owns stock or has equity interest (outside of mutual funds or other externally directed accounts) in any pharmaceutical or biotechnology company. TLSB has investigator‐initiated research funding from the NIH, the Alzheimer's Association, the Barnes‐Jewish Hospital Foundation, and Avid Radiopharmaceuticals. Dr. Benzinger participates as a site investigator in clinical trials sponsored by Avid Radiopharmaceuticals, Eli Lilly and Company, Biogen, Eisai, Jaansen, and F. Hoffmann‐La Roche, Ltd. She also serves as an unpaid consultant to Eisai and Siemens and is on the speaker's bureau for Biogen. AER reports no competing interests. He receives research support for this work from the National Institute on Aging (R01AG053267, U19AG032438). TI reports no competing interests. He received research support for this work from AMED (JP23dk0207066 and JP23dk0207049). GSD reports no competing interests directly relevant to this work. His research is supported by NIH (K23AG064029, U01AG057195, U01NS120901, U19AG032438). He serves as a consultant for Parabon Nanolabs Inc and as a topic editor (Dementia) for DynaMed (EBSCO). He is the co‐project PI for a clinical trial in anti‐NMDAR encephalitis, which receives support from Amgen Pharmaceuticals, and a consultant for Arialys Therapeutics. He has developed educational materials for PeerView Media, Inc., and Continuing Education Inc. He owns stock in ANI pharmaceuticals. Dr. Day's institution has received support from Eli Lilly for development and participation in an educational event promoting early diagnosis of symptomatic Alzheimer's disease, and in‐kind contributions of radiotracer precursors for tau‐PET neuroimaging in studies of memory and aging (via Avid Radiopharmaceuticals, a wholly owned subsidiary of Eli Lilly). RJP is Neuropathology Core Leader for the DIAN observational study and the DIAN Trials Unit. He receives research support for this work from the National Institute on Aging (U19 AG032438, U19AG032438‐09S1, R01AG068319). His laboratory receives cost recovery funding from Biogen for tissue procurement and processing services related to ALS clinical trials. Neither he nor his family owns stock or has equity interest (outside of mutual funds or other externally directed accounts) in any pharmaceutical or biotechnology company. FL has grants not related to this paper from NIH, DIAN, Enroll‐HD and BIOGEN. JL reports speaker fees from Bayer Vital, Biogen, EISAI, TEVA, Zambon, Esteve, Merck, and Roche; consulting fees from Axon Neuroscience, EISAI, and Biogen; author fees from Thieme medical publishers and W. Kohlhammer GmbH medical publishers; and is inventor in a patent “Oral Phenylbutyrate for Treatment of Human 4‐Repeat Tauopathies” (EP 23 156 122.6) filed by LMU Munich. In addition, he reports compensation for serving as chief medical officer for MODAG GmbH, is beneficiary of the phantom share program of MODAG GmbH, and is inventor in a patent “Pharmaceutical Composition and Methods of Use” (EP 22 159 408.8) filed by MODAG GmbH, all activities outside the submitted work. SBB receives support from the National Institute on Aging (NIA) and the Michael J. Fox Foundation. All other authors have nothing to disclose.

## DIAN CONTRIBUTIONS AND DATA HANDLING

This project's data collection and dissemination were supported by The Dominantly Inherited Alzheimer Network (DIAN, U19AG032438), funded by the National Institute on Aging (NIA), along with contributions from the Alzheimer's Association (SG‐20‐690363‐DIAN), the German Center for Neurodegenerative Diseases (DZNE), Raul Carrea Institute for Neurological Research (FLENI), and other international entities. These include partial funding from Japan's Agency for Medical Research and Development (AMED: JP23dk0207066 and JP23dk0207049), and support from the Korea Dementia Research Project through the Korea Dementia Research Center (KDRC), funded by the Ministry of Health & Welfare and Ministry of Science and ICT, Republic of Korea (HU21C0066), the Spanish Institute of Health Carlos III (ISCIII), the Canadian Institutes of Health Research (CIHR), the Canadian Consortium of Neurodegeneration and Aging, the Brain Canada Foundation, and the Fonds de Recherche du Québec—Santé. The DIAN Study investigators have vetted this manuscript for scientific accuracy and consistency with past publications. We are deeply grateful to our participants and their families for their generosity, as well as to the dedicated DIAN research and support staff across all sites.

## DIAN DATA ACCESSIBILITY

Due to the rarity of dominantly inherited Alzheimer's disease, individual‐level data from DIAN cannot be shared publicly, as it would compromise participant anonymity. This limitation has been validated by the institutional review board (IRB) and confirmed with the NIH. Nevertheless, this data remains accessible for qualified researchers upon request. Requests can be submitted through the following link: DIAN Biospecimen Request Form.

## CONSENT STATEMENT

All procedures were approved by the institutional review board at Washington University in St. Louis. Written informed consent was obtained from participants or their caregivers, adhering to the guidelines of their respective local institutional review boards.

## Supporting information



Supporting information

Supporting information

## DIAN CONSORTIUM LIST

Randall J. Bateman, James M. Noble, Gregory S. Day, Neill R. Graff‐Radford, Jonathan Vöglein, Ricardo Allegri, Patricio Chrem Mendez, Ezequiel Surace, Sarah B. Berman, Snezana Ikonomovic, Neelesh Nadkarni, Francisco Lopera, Laura Ramirez, David Aguillon, Yudy Leon, Claudia Ramos, Diana Alzate, Ana Baena, Natalia Londono, Sonia Moreno, Mathias Jucker, Christoph Laske, Elke Kuder‐Buletta, Susanne Graber‐Sultan, Oliver Preische, Anna Hofmann, Takeshi Ikeuchi, Kensaku Kasuga, Yoshiki Niimi, Kenji Ishii, Michio Senda, Raquel Sanchez‐Valle, Pedro Rosa‐Neto, Nick Fox, Dave Cash, Jae‐Hong Lee, Jee Hoon Roh, Meghan Riddle, William Menard, Courtney Bodge, Mustafa Surti, Leonel Tadao Takada, Martin Farlow, Jasmeer P. Chhatwal, V. J. Sanchez‐Gonzalez, Maribel Orozco‐Barajas, Alison Goate, Alan Renton, Bianca Esposito, Celeste M. Karch, Jacob Marsh, Carlos Cruchaga, Victoria Fernandez, Brian A. Gordon, Anne M. Fagan, Gina Jerome, Elizabeth Herries, Jorge Llibre‐Guerra, Allan I. Levey, Erik C. B. Johnson, Nicholas T. Seyfried, Peter R. Schofield, William Brooks, Jacob Bechara, Eric McDade, Jason Hassenstab, Richard J. Perrin, Erin Franklin, Tammie L. S. Benzinger, Allison Chen, Charles Chen, Shaney Flores, Nelly Friedrichsen, Nancy Hantler, Russ Hornbeck, Steve Jarman, Sarah Keefe, Deborah Koudelis, Parinaz Massoumzadeh, Austin McCullough, Nicole McKay, Joyce Nicklaus, Christine Pulizos, Qing Wang, Sheetal Mishall, Edita Sabaredzovic, Emily Deng, Madison Candela, Hunter Smith, Diana Hobbs, Jalen Scott, Johannes Levin, Chengjie Xiong, Peter Wang, Xiong Xu, Yan Li, Emily Gremminger, Yinjiao Ma, Ryan Bui, Ruijin Lu, Ralph Martins, Ana Luisa Sosa Ortiz, Alisha Daniels, Laura Courtney, Hiroshi Mori, Charlene Supnet‐Bell, Jinbin Xu, John Ringman.
