# Supplementary material for: Ubiquitin–proteasome system in the different stages of dominantly inherited Alzheimer's disease
Source: Alzheimers Dement. 2025 May 24;21(5):e70243. doi: 10.1002/alz.70243 (PMC12102666; doi:10.1002/alz.70243)
Supplement: Supplementary file 1 — Supporting information [file ALZ-21-e70243-s002.docx]

**Supplementary Figure 1:** Estimated Effects of UPS Proteins on Baseline Cognitive Performance


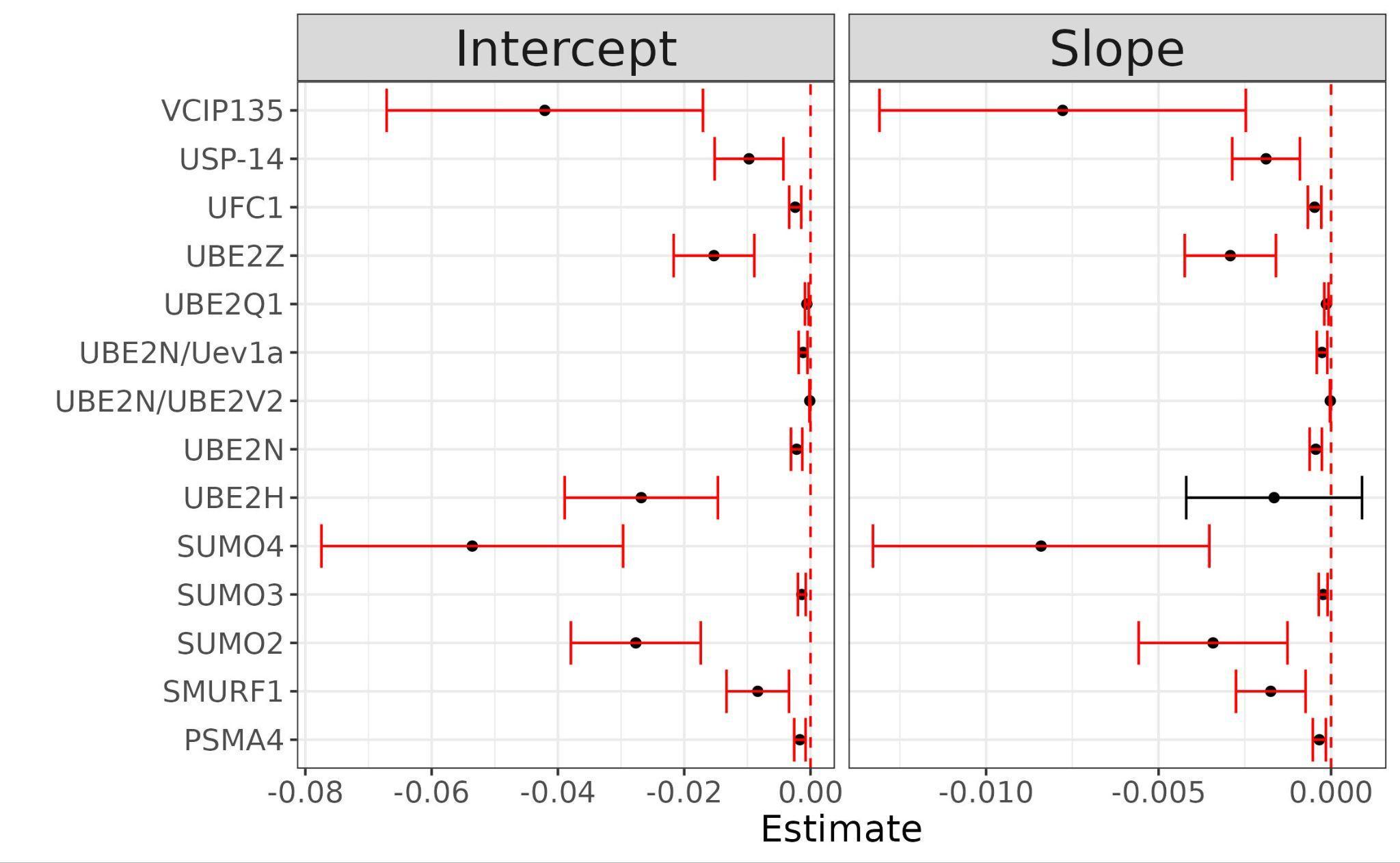


Supplementary Figure 1: This figure displays the 95% confidence intervals for the effects of each UPS protein on both the intercept and slope of the cognitive composite score. The detailed estimation results are provided in the corresponding tables. To account for multiple comparisons, FDR-adjusted p-values were calculated, and proteins with FDR-adjusted p-values below 0.05 are highlighted in red. Intercept Effect: Represents the change in the baseline cognitive composite score associated with a one-unit increase in protein level. Slope Effect: Represents the impact of a one-unit increase in protein level on the annual rate of cognitive decline.

**Supplementary Figure 2:** UPS protein levels across A/T Biological Stages


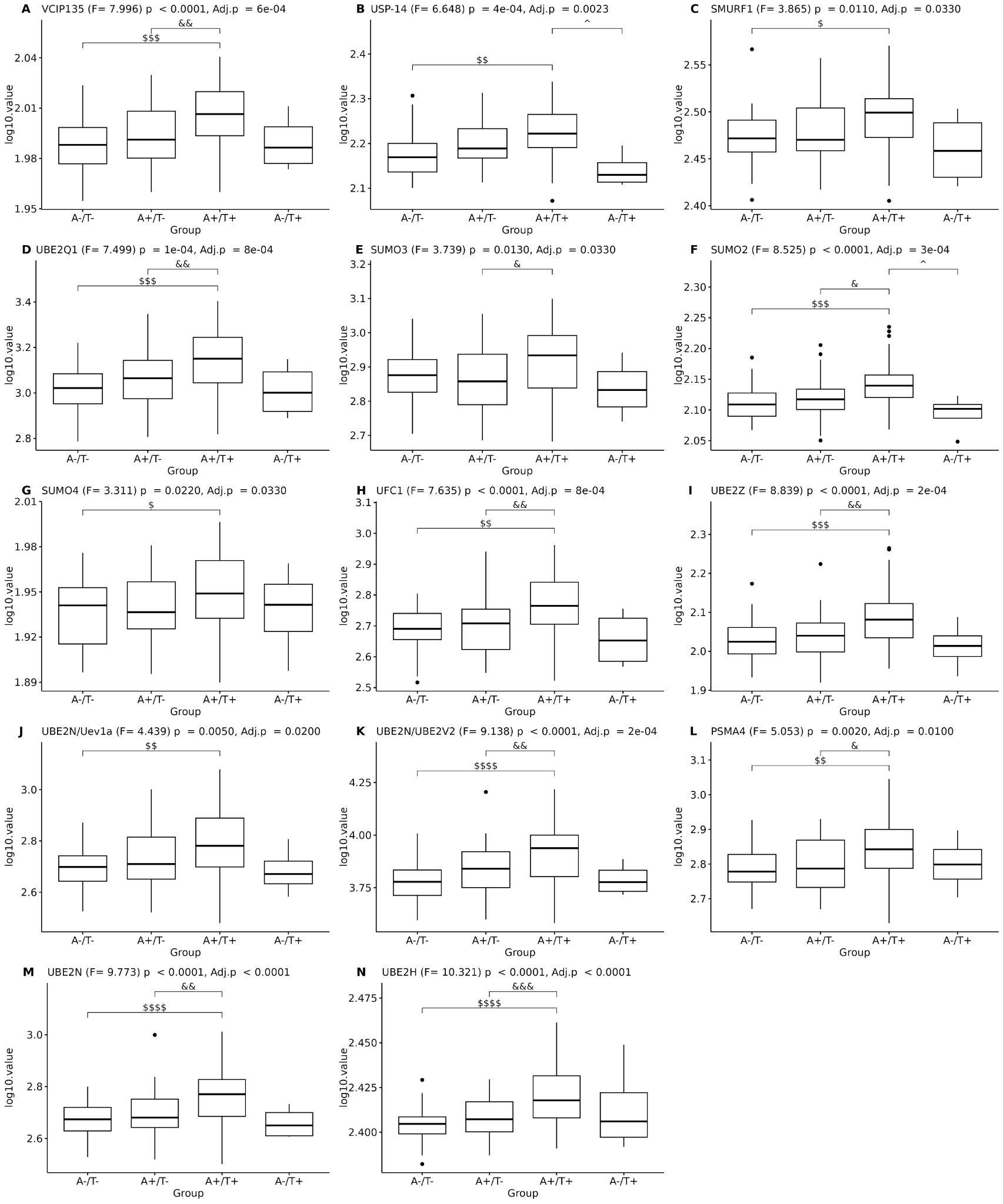


Supplementary Figure 2:The box plots illustrate the log₁₀-transformed protein levels of 14 UPS proteins across four Amyloid PET/(PT205/T205) (A/T) classification groups: A-/T-, A+/T-, A+/T+, and A-/T+. The x-axis represents the A/T groups, while the y-axis denotes the log₁₀-transformed protein levels. Statistical significance was assessed using ANOVA for overall differences, followed by Tukey’s HSD test for pairwise comparisons.

The p-values for each protein are indicated in each plot, with significant pairwise differences marked by different symbols, representing different levels of significance. A represents Amyloid PET, T represents the PT205/T205 ratio, PT205 refers to phosphoTau205, and T205 refers to total Tau205. Statistical significance is denoted as follows: ^, &, $ for p < 0.05; ^^, &&, $$ for p < 0.001; ^^^, &&&, $$$ for p < 0.0001; and ^^^^, &&&&, &$$$ for p < 0.00001.

**Supplementary Figure3**:Autolysosomal Pathway Protein Levels Relative to EYO at Baseline


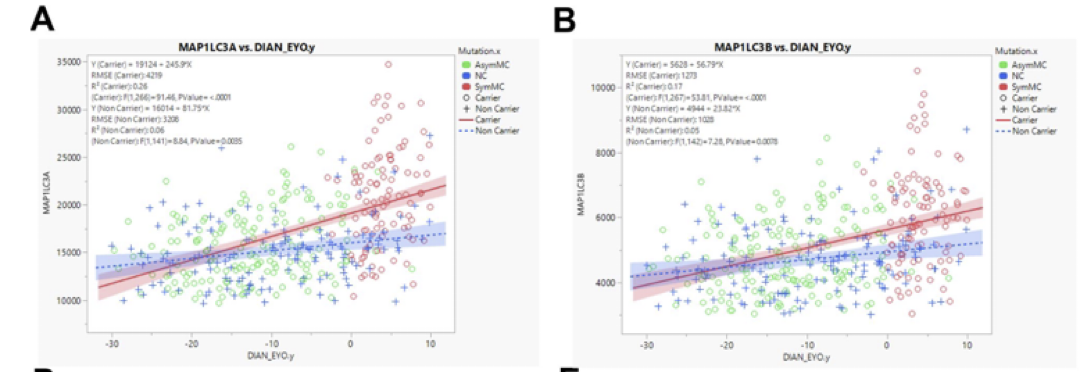


Supplementary Figure 3: Association between MAP1LC3A (A) and MAP1LC3B (B) expression levels and estimated years of symptom onset (DIAN_EYO) among individuals with different mutation statuses. Each data point represents an individual and is color-coded by mutation status and disease stage: Mutation Carriers (red line) and Non-Mutation Carriers (blue line) for various genetic mutations. ​The analysis demonstrates that MAP1LC3 protein levels are statistically significantly elevated in mutation carriers compared to non-mutation carriers.​ In the scatter plots, blue crosses indicate non-mutation carriers, green circles represent asymptomatic mutation carriers, and red circles denote symptomatic mutation carriers, illustrating the distinct expression patterns associated with disease progression.

**Supplementary Table 1:** Sensitivity Analysis of UPS Protein Slopes Over DIAN EYO Excluding Symptomatic Mutation Carriers

| UPS | Slope Estimate  ( MC vs NC) | SE | Lower  ( 95%CI) | Upper  ( 95% CI) | P value  (FDR p) |
| --- | --- | --- | --- | --- | --- |
| VCIP135 | 0.0545 | 0.0469 | -0.0373 | 0.1464 | 0.2454 |
| USP-14 | 0.3531 | 0.2381 | -0.1136 | 0.8198 | 0.1392 |
| SMURF1 | 0.2861 | 0.2186 | -0.1425 | 0.7146 | 0.1916 |
| UBE2Q1 | 1.9483 | 3.6358 | -5.1777 | 9.0743 | 0.5924 |
| SUMO3 | 0.0580 | 1.7399 | -3.3521 | 3.4681 | 0.9734 |
| SUMO2 | 0.0701 | 0.0959 | -0.1178 | 0.2580 | 0.4652 |
| SUMO4 | -0.0137 | 0.0424 | -0.0969 | 0.0694 | 0.7466 |
| UFC1 | 0.1862 | 1.0942 | -1.9583 | 2.3307 | 0.8650 |
| UBE2Z | 0.0944 | 0.1410 | -0.1819 | 0.3707 | 0.5034 |
| UBE2N/Uev1a | 0.7267 | 1.5490 | -2.3093 | 3.7628 | 0.6393 |
| UBE2N/UBE2V2 | 22.3637 | 20.6232 | -18.0571 | 62.7845 | 0.2790 |
| PSMA4 | 0.3014 | 1.0980 | -1.8505 | 2.4534 | 0.7838 |
| UBE2N | 1.2739 | 1.0858 | -0.8542 | 3.4020 | 0.2415 |
| UBE2H | 0.0627 | 0.0835 | -0.1010 | 0.2264 | 0.4531 |

Supplementary Table 1: This table presents slope estimates from linear regression analyses of UPS protein levels plotted against DIAN-EYO at baseline, after excluding symptomatic mutation carriers (MCs). The slope (β) for each protein, standard error (SE), 95% confidence interval, and p-values (raw and FDR-adjusted) are provided.

### **Supplementary Table 2.** Estimated Intercept and Slope Effects of 14 UPS Proteins on Cognitive Performance

| Effect Type | Variable | Estimate | STE | Lower  (95% CI) | Upper  (95% CI) | P value  (FDR p) |
| --- | --- | --- | --- | --- | --- | --- |
| Intercept | VCIP135 | -0.042943 | 0.012604 | -0.067784 | -0.018102 | 0.000912 |
| Intercept | USP-14 | -0.010205 | 0.002733 | -0.015596 | -0.004814 | 0.000386 |
| Intercept | SMURF1 | -0.008106 | 0.002492 | -0.013018 | -0.003194 | 0.001428 |
| Intercept | UBE2Q1 | -0.000561 | 0.000152 | -0.000861 | -0.000260 | 0.000414 |
| Intercept | SUMO3 | -0.001360 | 0.000315 | -0.001980 | -0.000740 | 0.000041 |
| Intercept | SUMO2 | -0.027982 | 0.005170 | -0.038171 | -0.017793 | 0.000002 |
| Intercept | SUMO4 | -0.052501 | 0.012024 | -0.076198 | -0.028803 | 0.000039 |
| Intercept | UFC1 | -0.002378 | 0.000484 | -0.003331 | -0.001425 | 0.000008 |
| Intercept | UBE2Z | -0.015375 | 0.003213 | -0.021705 | -0.009044 | 0.000009 |
| Intercept | UBE2N/Uev1a | -0.001122 | 0.000353 | -0.001818 | -0.000426 | 0.001705 |
| Intercept | UBE2N/UBE2V2 | -0.000117 | 0.000024 | -0.000165 | -0.000070 | 0.000008 |
| Intercept | PSMA4 | -0.001648 | 0.000458 | -0.002550 | -0.000745 | 0.000502 |
| Intercept | UBE2N | -0.002187 | 0.000451 | -0.003074 | -0.001299 | 0.000008 |
| Intercept | UBE2H | -0.028780 | 0.006119 | -0.040840 | -0.016721 | 0.000011 |
| Slope | VCIP135 | -0.007928 | 0.002646 | -0.013196 | -0.002660 | 0.003953 |
| Slope | USP-14 | -0.001866 | 0.000483 | -0.002829 | -0.000903 | 0.000563 |
| Slope | SMURF1 | -0.001770 | 0.000504 | -0.002770 | -0.000769 | 0.001053 |
| Slope | UBE2Q1 | -0.000134 | 0.000031 | -0.000195 | -0.000073 | 0.000080 |
| Slope | SUMO3 | -0.000228 | 0.000064 | -0.000356 | -0.000101 | 0.000986 |
| Slope | SUMO2 | -0.003402 | 0.001075 | -0.005540 | -0.001263 | 0.002531 |
| Slope | SUMO4 | -0.008378 | 0.002432 | -0.013215 | -0.003541 | 0.001258 |
| Slope | UFC1 | -0.000473 | 0.000098 | -0.000667 | -0.000279 | 0.000020 |
| Slope | UBE2Z | -0.002885 | 0.000662 | -0.004196 | -0.001575 | 0.000080 |
| Slope | UBE2N/Uev1a | -0.000257 | 0.000076 | -0.000408 | -0.000106 | 0.001281 |
| Slope | UBE2N/UBE2V2 | -0.000023 | 0.000005 | -0.000033 | -0.000014 | 0.000020 |
| Slope | PSMA4 | -0.000337 | 0.000095 | -0.000525 | -0.000149 | 0.000986 |
| Slope | UBE2N | -0.000440 | 0.000089 | -0.000616 | -0.000265 | 0.000020 |
| Slope | UBE2H | -0.001659 | 0.001268 | -0.004171 | 0.000853 | 0.193239 |

Supplementary Table 2: This table details the estimated effects of each UPS protein on baseline cognitive scores (intercept) and annual cognitive decline (slope), corresponding to Supplementary Figure 1. Values include estimates, 95% confidence intervals, and FDR-adjusted p-values.
